# A Semi-Mechanistic Mathematical Model of Immune Tolerance Induction to Support Preclinical Studies of Human Monoclonal Antibodies in Rats

**DOI:** 10.3390/pharmaceutics17070845

**Published:** 2025-06-27

**Authors:** Paridhi Gupta, Josiah T. Ryman, Vibha Jawa, Bernd Meibohm

**Affiliations:** 1 Department of Pharmaceutical Sciences, University of Tennessee Health Science Center, Memphis, TN 38163, USA; pgupta7@uthsc.edu; 2 EMD Serono Research and Development Institute, Billerica, MA 01821, USA; 3 Clinical Pharmacology, Pharmacometrics and Bioanalysis, Bristol Myers Squibb, Princeton, NJ 08543, USA

**Keywords:** monoclonal antibodies, preclinical, pharmacokinetics, anti-drug antibodies, semi-mechanistic model, population pharmacokinetic model, immune tolerance

## Abstract

**Background/Objectives:** The administration of human monoclonal antibodies (mAb) in preclinical pharmacokinetics and toxicology studies often triggers an immune response, leading to the formation of anti-drug antibodies (ADA). To mitigate this effect, we have recently performed and reported on studies using short-term immunosuppressive regimens to induce prolonged immune tolerance towards a human mAb, erenumab, in rats. Here, we report on the development of a semi-mechanistic modeling approach that quantitatively integrates pharmacokinetic and immunogenicity assessments from immune tolerance induction studies to provide a framework for the simulation-based evaluation of different immune induction scenarios for the maintenance of prolonged immune tolerance towards human mAbs. **Methods:** The integrated pharmacokinetic/pharmacodynamic (PK/PD) modeling approach combined a semi-mechanistic model of the adaptive immune system to predict ADA formation kinetics with a population pharmacokinetic model to assess the impact of the time course of the ADA magnitude on the PK of erenumab in rats. Model-derived erenumab concentration–time profiles served as input for a quantitative system pharmacology-style semi-mechanistic model of the adaptive immune system to conceptualize the ADA response as a function of the kinetics of CD4^+^ T helper cells and T regulatory cells. **Results:** The model adequately described the observed ADA magnitude–time profiles in all treatment groups and reasonably simulated the kinetics of selected immune cells responsible for ADA formation. It also successfully captured the impact of tacrolimus/sirolimus immunomodulation on ADA formation, demonstrating that the regimen effectively suppressed ADA formations and induced immune tolerance. **Conclusions**: This work demonstrates the utility of modeling approaches to integrate pharmacokinetic and immunogenicity assessment data for the prospective planning of long-term toxicology studies to support the preclinical development of mAbs.

## 1. Introduction

Over the past decade, there has been a significant increase in the number of approved therapeutic proteins (TP), such as monoclonal antibodies (mAbs), for human use, with many more currently in preclinical and clinical development. Most mAbs have been engineered to be either identical or close to endogenous human immunoglobulins [1]. Therefore, they often have limited immunogenicity in humans; however, immunogenicity is observed with high frequency when administered to animal species during preclinical drug development [2]. Immunogenicity, particularly the induction of anti-drug antibodies (ADA), has the potential to become a challenge and liability during drug development due to its impact on drug pharmacokinetics (PK) and systemic exposure, as it can lead to the formation of immune complexes with the TP and subsequent immune complex-mediated clearance [3,4].

The development of ADA involves complex immunological mechanisms such as antigen presentation, activation of T and B immune cells, and cytokine production and release [5]. To assess the immunogenicity of TP, several approaches have been explored. These include the use of T cell or B cell epitope prediction methods based on protein sequence or structure [6,7,8,9,10,11], in vitro major histocompatibility complex (MHC)–peptide binding assays [12,13], and T cell proliferation assays [14,15]. The complicated biological processes involved and a large number of factors impacting ADA formation (e.g., amino acid sequences, aggregates, impurities, and co-medication) make it difficult to quantitatively predict ADA responses and effects [16,17]. As a result, semi-mechanistic mathematical models have been applied more recently to serve as a complementary approach to ADA predictions, because the underlying biology of the adaptive immune response to an antigenic TP that can result in ADA formation has been well characterized and can be integrated into these modeling approaches [18].

In 2014, Chen et al. published a multiscale mechanistic model of the adaptive immune response to predict ADA formation for TP and applied the model to predict the immunogenicity of adalimumab [16,19]. Further, in 2019, Hamuro et al. updated the work published by Chen et al. and utilized it to predict ADA incidence for an Fc-fusion protein in clinical development [20]. These models are unique compared to previously used limited modeling approaches for immunogenicity, as they link protein sequence-based T cell epitope prediction methods to downstream processes central to the immune response: antigen presentation and the T and B cell biology required for ADA generation [16,20,21]. When combined with a drug disposition model, they can also be used to predict the impact of ADA formation on the PK of the affected TP [16,20].

The impact of ADA on the PK and systemic exposure of TPs is particularly an issue in the preclinical drug development of TPs, where ADA may prevent toxicology studies from establishing safe exposure ranges of the TP in study animals [22]. This may confound and limit the selection of a safe starting dose for first-in-human clinical trials [2,23,24].

Therefore, the objective of this work was to develop and validate a semi-mechanistic model of the adaptive immune system to characterize differences in ADA formation under different scenarios of drug-induced immunosuppression and explore the persistence of induced immune tolerance for a fully human mAb, erenumab, during chronic toxicology studies in rats. Here, we developed a population pharmacokinetic model to characterize the impact of ADA formation on the PK of erenumab, where mAb was administered at different dose levels, in different aggregation states, and with or without concomitant immunosuppressants. The PK model was integrated with a semi-mechanistic adaptive immune system model of immune tolerance development, which was a simplified version of the model structures proposed by Chen et al. and Hamuro et al. to predict selected immune cell kinetics involved in downstream ADA production and the resulting time courses of ADA formation with the help of drug-specific and species-specific parameters. The model accommodated the differential effects of the tested immunosuppressive drugs and confirmed that a tacrolimus/sirolimus combination is superior in inducing prolonged immune tolerance to erenumab compared to methotrexate regimens. The final model was utilized to simulate a hypothetical 6-month chronic toxicology study to explore the persistence of immune tolerance towards erenumab induced by the short-term administration of the tacrolimus/sirolimus combination, thereby highlighting its potential future application in the drug development environment.

## 2. Materials and Methods

### 2.1. Studies Providing PK and ADA Data

Our analysis was based on the results from two preclinical studies in Sprague Dawley rats that investigated the effect of the short-term administration of immunosuppressive drug regimens on the induction and maintenance of immune tolerance towards a human monoclonal antibody, erenumab. The design, analysis, and results of these studies are described elsewhere in detail [25].

In brief, two immune tolerance studies were conducted, each over a period of 12 weeks. Both studies were divided into three phases: an induction phase (weeks 1–4), a washout phase (weeks 5–8), and a rechallenge phase (weeks 9–12). During the induction phase, animals received weekly subcutaneous (SC) doses of erenumab, fully human immunoglobulin G (IgG2) mAb, along with concurrent immunosuppressive therapy. While erenumab is designed to block the calcitonin gene-related peptide receptor in humans, it does not bind to any specific target in rats. In the washout phase, animals did not receive erenumab to ensure a complete washout of the antibody, but the immunosuppressive therapy was continued until week 7 of the study. In the rechallenge phase, animals were administered erenumab weekly but without any concurrent immunosuppression. In Study 1, a weekly erenumab dose of 10 mg/kg was administered in monomeric form, and in Study 2, 1 mg/kg erenumab per week was administered in an aggregate form.

Study 1 consisted of four groups (n = 8 per group). Animals in Group 1.1, the control group, received erenumab without any immunosuppression, while animals in the other three groups received erenumab in combination with immunosuppressive drug regimens. Animals in Group 1.2 (MTX (5 mg/kg) + erenumab monomer) were treated with erenumab monomer and methotrexate (5 mg/kg) administered intraperitoneally once every week from week 1 to week 7. Animals in Group 1.3 (MTX (3 mg/kg) + erenumab monomer) received three intraperitoneal injections of methotrexate (3 mg/kg) on the first, second, and third day in week 1. Animals in Group 1.4 (TAC/SIR + erenumab monomer) were administered sirolimus (6 mg/kg) by oral gavage on the first day of the study and tacrolimus (2 mg/kg SC) once weekly from week 1 to week 7.

**Figure 1:**
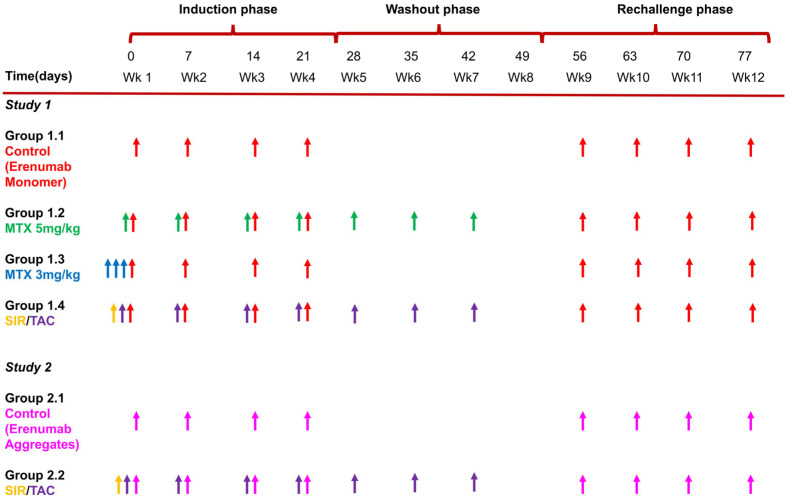
Schematic representation of the study design and dosing strategy in all treatment groups of Study 1 and Study 2. Arrows indicate different dosing events: Red: Erenumab monomer; Pink: Erenumab aggregate; Green: MTX 5 mg/kg; Blue: MTX 3 mg/kg; Yellow: Sirolimus; Purple Tacrolimus. From [25].

Study 2 consisted of two groups (n = 10 per group). Animals in Group 2.1 received erenumab without any immunosuppression, while animals in Group 2.2 (TAC/SIR + erenumab aggregates) received treatment with the same tacrolimus/sirolimus regimen as in Group 1.4. The study design for both studies is summarized in Figure 1.

All animal experimentation was conducted in accordance with the Animal Welfare Act and the Public Health Service Policy on Humane Care and Use of Laboratory Animals. Prior to initiation, all animal protocols were approved by the Institutional Animal Care and Use Committees of the University of Tennessee Health Science Center.

Serum samples were obtained from all animals throughout the study duration at 12 and 36 pre-defined time points for ADA and erenumab quantification, respectively. The relative amount of ADA in the collected specimens was quantified with an acid-dissociation bridging electrochemiluminescence immunoassay (ECLIA), and ADA magnitude in each sample was expressed as a signal-to-noise ratio (S/N). A S/N ratio of greater than 2 and 1.5 was set as a positive ADA measurement for Study 1 and Study 2, respectively. The assay could tolerate 40 μg/mL of excess erenumab in the presence of 100 ng/mL of the donkey anti-human IgG antibody. Erenumab concentrations were quantified with an ECLIA-based assay with a calibration range of 0.078–10 μg/mL. Further details on the sampling strategy and the bioanalytical assays can be found in the Supplementary Material.

### 2.2. Model Development

#### 2.2.1. Population Pharmacokinetic Model

In the first model-building step, a population pharmacokinetic model was developed that describes the impact of ADA formation and erenumab’s aggregation state on the pharmacokinetics of erenumab in rats.

##### Structural Model

A structural PK model was developed from erenumab concentration data from ADA-negative and ADA-positive animals in Study 1 and Study 2. In the first step, 1406 erenumab concentrations from 38 ADA-negative animals were utilized, and different inter-individual variability (IIV), inter-occasion variability (IOV), and residual error models were evaluated. Further, the effect of covariates on the PK parameters was assessed, including body weight and the erenumab aggregation state. Once the structural model was finalized, in the second step, an additional 518 erenumab concentrations from 14 ADA-positive animals were incorporated to inform the full model, and the impact of ADA as a covariate on the PK of erenumab was also evaluated.

**Figure 2:**
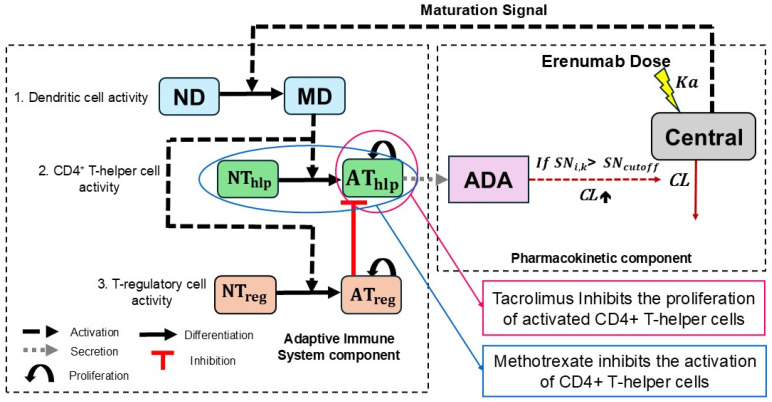
Schematic representation of the pharmacokinetic and semi-mechanistic immune cell dynamics model structure (PK/ADA model). The acronyms are explained as follows: central: central compartment; CL: linear drug clearance; ADA: anti-drug antibody; SN_i,k_: signal:noise ratio (ADA magnitude) for ith animal at time k; SN_cutoff_: threshold of ADA magnitude beyond which the sample is considered ADA-positive; ND: naïve dendritic cell; MD: mature dendritic cell; NT_hlp_: naïve CD4^+^ T helper cell; AT_hlp_: activated CD4^+^ T helper cell; NT_reg_: naïve T regulatory cell; AT_reg_: activated T regulatory cell.

One- and two-compartment models with first-order absorption, distribution, and elimination processes were tested. Competing structural models were compared using Akaike’s information criterion (AIC), defined as follows: AIC = OFV + (2 × NPR), where NPR is the total number of parameters estimated in each model, and OFV is the objective function value of the model fit. The model with the lowest AIC and OFV value was selected as the base model for PK analysis. A one-compartment model that estimated PK parameters, first-order absorption rate constant (Ka), apparent volume of distribution (V_d_/F), and apparent clearance (CL/F) was sufficient to describe the observed data. Initial PK parameter estimates for model development were taken from a previously published study that also evaluated the PK of a human mAb in rats [26]. Parameters V_d_/F and CL/F were considered apparent because erenumab was administered extravascularly by SC injection, and bioavailability F remained unknown. The model structure is shown in the pharmacokinetic component of Figure 2.

##### Inter-Individual Variability, Inter-Occasion Variability, and Residual Error Model

IIV and IOV in the PK parameters were described using an exponential model depicted as follows:

(1) $$\theta_{i,j}=\;\theta_{pop}\cdot \;e^{{ɳ}_{i}}\cdot \;e^{\gamma_{i,j}},$$

where θ_i,j_ is the estimated parameter for subject i on occasion j, θ_pop_ is the typical population value of the parameter, and η_i_ is the random effect for individual i accounting for IIV, which is assumed to be normally distributed with a mean of zero and variance of ω^2^_IIV_. γ_i,j_ is the occasion-specific random effect for individual i on occasion j, which is assumed to be normally distributed with a mean of zero and variance of ω^2^_IOV_.

IIV was tested on all model parameters. IOV with each dosing interval defined as an occasion was tested specifically on Ka, as the erenumab injection site was rotated from dose to dose between multiple subcutaneous regions in the same animal. Eight distinct occasions were defined based on the eight erenumab injection time points during the induction and the rechallenge phase. IIV and IOV for the identified PK parameters were assessed independently for Study 1 and Study 2. Additive, proportional, and combined additive and proportional residual error models were tested, with separate error models evaluated for Study 1 and Study 2.

IIV, IOV, and residual error models were explored independently for the two studies because different bioanalytical assays, different instruments used for sample analysis, study conditions (e.g., seasonal factors), different batches of erenumab, and different animal populations may have affected random variability in the two separate studies that were performed 15 months apart [27].

##### Covariates

During PK model development, several covariates were evaluated. The covariates tested were body weight, aggregation state of erenumab, and ADA magnitude. Weight was evaluated as a continuous, time-varying covariate on CL/F and V_d_/F, centered around the median weight of all study animals. The structure of the covariate model is shown below.

(2) $$\theta_{i}=\;\theta_{pop}\cdot {\frac{{WT}_{i}}{{WT}_{ref}}}^{\beta_{weight}},$$

where WT_i_ is the weight of the individual animal, WT_ref_ is the median weight of the animals in the study, and β_weight_ is the allometric scaling exponent.

The aggregation state of erenumab (monomer versus aggregates) was assessed as a binary covariate for CL/F, V_d_/F, and Ka to quantify how aggregation affects the PK of erenumab and was implemented as indicated below:

(3) $$\theta_{i}=\;\theta_{pop}\cdot \;{\theta_{aggregation}}^{state}\;,$$

where the parameter estimate for the effect of aggregation on a PK parameter (θ_aggregation_) was raised to the indicator variable for the erenumab aggregation state coded as 0 or 1 depending on whether erenumab was administered as monomer or aggregate, respectively.

ADA was incorporated as a continuous, time-varying covariate for CL/F, where the ADA effect was driven by its magnitude (quantified as S/N) at each of the 12 observation time points [28]. The covariate model was mathematically expressed as follows:

(4) $${CL}_{i,k}=\;{CL}_{pop}{\cdot \frac{{SN}_{i,k}}{{SN}_{cut-off}}}^{\theta_{ADA}};if\;{SN}_{i,k}>\;{SN}_{cutoff}\;and\;\;{CL}_{i,k}=\;{CL}_{pop}\;;if\;{SN}_{i,k}\leq \;{SN}_{cutoff,}$$

where CL_i,k_ is the clearance of the ith animal at time k, CL_pop_ is the typical population value of clearance, SN_i,k_ is the observed ADA magnitude for the ith animal at time k, SN_cutoff_ is the threshold of ADA magnitude beyond which the multiplicative factor to quantify the increase in clearance could be applied, and θ_ADA_ is the exponent in ADA effect. θ_ADA_ is the parameter estimated upon fitting the model to the observed ADA S/N, which describes the saturable non-linear relationship between ADA magnitude and clearance.

The influence of each covariate on the PK parameter was tested using the likelihood ratio χ^2^ test with a significance level (α) of 0.05 (change in OFV ≥ 3.84) and a reduction in IIV of 10% or more. The covariates remained in the final model if their addition significantly improved the model fit.

##### PK Model Evaluation, Goodness-of-Fit, and Model Qualification

The development of the population PK model was guided by a review of standard goodness-of-fit plots, a significant reduction in OFV, the plausibility of the parameter estimates, and previous knowledge of mAb PK parameters from published preclinical and clinical studies.

For model qualification, a visual predictive check (VPC) and a non-parametric bootstrap analysis were conducted. In the VPC, the final model was used to perform Monte Carlo-type simulations of the dataset used for model development, with 1000 replicates, and the median and 90% prediction interval constructed from the simulated erenumab concentration–time profiles were compared with the observed data. VPC was stratified according to the ADA status of the animal (ADA-positive versus ADA-negative) and dose/aggregation state (10 mg/kg erenumab monomer in Study 1 versus 1 mg/kg erenumab aggregate in Study 2). Likewise, a non-parametric bootstrap analysis was executed with 1000 replicates to assess the precision and robustness of the final parameter estimates. The median and 90% confidence interval for each parameter were constructed from the parameter distributions obtained from the bootstrap runs.

The final population PK model was used to inform individual animal PK parameters, which served as input for the development of the semi-mechanistic model in Step 2.

#### 2.2.2. Semi-Mechanistic Immune Cell Dynamics Model

In the second model-building step, a semi-mechanistic immune cell dynamics model of the adaptive immune system was developed to describe the induction and maintenance of immune tolerance against erenumab in rats.

##### Structural Model

In this component, a semi-mechanistic model of the adaptive immune system was developed that captures key processes involved in the activation, differentiation, and proliferation of selected immune cell populations in response to erenumab exposure that can ultimately mimic the differential effects on downstream ADA production. The model was evaluated for its ability to predict the impact of immunosuppressive drug-induced immune tolerance on ADA kinetics.

The goal behind adopting a semi-mechanistic modeling approach was driven by the need to understand the cellular-level dynamics, specifically how the various immune cell populations interact and change to influence ADA formation in animals under different treatment conditions. In addition, the immune tolerance effects induced by methotrexate and tacrolimus/sirolimus on ADA formation could not have been predicted using traditional empirical modeling methods such as population PK modeling that treat ADA as a covariate on drug clearance and thus lacks the upstream mechanistic details of immune cell activation and proliferation that are needed to predict the effects of concomitant immunosuppressive therapy [20].

We utilized, adapted, and simplified the work published by Chen et al. and Hamuro et al. [16,19,20]. Our model components comprised antigen-presenting dendritic cells, CD4^+^ T helper cells, and T regulatory cells as the major immune cell populations relevant for triggering an ADA production signal. Dendritic cell activation was modeled as being driven by a maturation signal (MS), which was assumed to be equal to the erenumab concentration (C) in the central compartment. The erenumab concentration–time profile for each individual animal was derived from the population PK model developed under item 2.2.1 according to Equation (5):

(5) $$\frac{dC}{dt}=\;\frac{K_{a}\cdot Dose}{V_{d}/F}-\frac{CL/F}{V_{d}/F}\cdot C,$$

where Dose is the administered erenumab dose, and Ka, V_d_/F, and CL/F are the erenumab pharmacokinetic parameters, as defined in the population PK analysis section.

The MS initiates the activation of naïve dendritic cells (ND), which then differentiate into mature dendritic cells (MD), as described by Equation (6). The first term represents the formation of MD cells from ND cells when encountering MS (represented by erenumab concentration C), while the second term accounts for the natural death of MD cells:

(6) $$\frac{dMD}{dt}=\;\left[\frac{\delta_{ND}\cdot C}{EC50+C}\right]\cdot ND-\;\beta_{MD}\cdot MD,$$

where δ_ND_ is the maximum activation rate constant for ND cells, EC50 is the erenumab concentration at which the ND cell activation rate is 50% maximum, and β_MD_ is the death rate constant for MD cells.

Further, MD cells serve as a connection between innate and adaptive immune systems, as they digest the antigenic erenumab into T epitopes. Subsequent recognition of CD4^+^ T helper epitope–MHC-II complexes and T regulatory epitope–MHC-II complexes in parallel on the MD cell surface via the T cell receptor facilitates the activation of naïve CD4^+^ T helper cells (NT_hlp_) and naïve T regulatory cells (NT_reg_), respectively [29]. Once activated, these naïve cells differentiate into corresponding activated CD4^+^ T helper cells (AT_hlp_) and activated T regulatory cells (AT_reg_), both of which proliferate. The activated CD4^+^ T helper cells were modeled as described in Equation (7): the first term shows the differentiation of AT_hlp_ cells from NT_hlp_ cells activated via interaction with MD cells, while the second term shows the proliferation of AT_hlp_ cells and AT_reg_ cells, inhibiting the proliferation rate of AT_hlp_ cells using the model of Velez de Mendizabal et al. [30], and the last term shows the natural death of AT_hlp_ Cells.

(7) $$\frac{d{AT}_{hlp}}{dt}=\;\delta_{{NT}_{hlp}}\cdot D\cdot {NT}_{hlp}+\rho_{{AT}_{hlp}}\cdot {AT}_{hlp}\cdot D\cdot \left[\frac{{AT}_{reg}50}{{(AT}_{reg}50+{AT}_{reg})}\right]-\;\beta_{{AT}_{hlp}}\cdot {AT}_{hlp},$$

where δ_NThlp_ is the maximum activation rate for NT_hlp_ cells, ρ_AThlp_ is the maximum proliferation rate for AT_hlp_ cells, AT_reg_50 is the number of AT_reg_ cells required for half-maximal suppression of AT_hlp_ cells, and β_AThlp_ is the death rate constant for AT_hlp_ cells.

The activated T regulatory cells were modeled as described in Equation (8): the first term shows the differentiation of AT_reg_ cells from NT_reg_ cells activated via interaction with MD cells, while the second term shows the proliferation of AT_reg_ cells, and the third term shows the natural death of AT_reg_ cells.

(8) $$\frac{d{AT}_{reg}}{dt}=\;\delta_{{NT}_{reg}}\cdot D\cdot {NT}_{reg}+\rho_{{AT}_{reg}}\cdot {AT}_{reg}\cdot D-\;\beta_{{AT}_{reg}}\cdot {AT}_{reg},$$

where δ_NTreg_ is the maximum activation rate for NT_reg_ cells, ρ_ATreg_ is the maximum proliferation rate for AT_reg_ cells, and β_ATreg_ is the death rate constant for At_reg_ cells.

(9) $$D=\;\frac{MD}{{MD+{NT}_{hlp}+{AT}_{hlp}+\;NT}_{reg}+{AT}_{reg}}.$$

Expression D depicted in Equation (9) accounts for the number of MD cells that are available for T cell activation. In general, a bigger T cell population will require more MD cells to activate the same proportion of cells since a single MD cell can only co-operate with a limited number of T cells at any time [31]. The function implies that the activation and proliferation of T cells by MD cells follow saturable kinetics, reflecting the finite capacity of MD cells to cooperate with T cells effectively.

The activated CD4^+^ T helper cell population was then linked as a driving force in ADA formation using the model of Ren et al. [32], thereby acknowleging that this is still a physiologically complex process involving B cells, plasma cells, and other immune system components. ADA formation was modeled as a zero-order process, with the rate of production of ADA being directly proportional to the number of AT_hlp_ cells. In addition, a lag time (T_lag_) was incorporated to account for the delay in ADA production, as shown below:

(10) $$ADA=\;\alpha \cdot \frac{{AT}_{hlp}}{K}\cdot \left[1-\;e^{{(-K}_{ADA}\cdot (t-T_{lag}))}\right]\mathrm{for}\;t\geq \;T_{lag}\;\;\;\mathrm{and}\;ADA=0\mathrm{for}\;t<\;T_{lag},$$

where α is the secretion rate of the antibody, AT_hlp_ is the number of activated CD4^+^ T helper cells, K_ADA_ is the elimination rate of the antibody, T_lag_ is the lag time for ADA formation, and K is a proportionality constant.

The model was expanded to also incorporate the immunosuppressive effect of the co-administration of either methotrexate or a tacrolimus/sirolimus combination. Methotrexate, a dihydrofolate reductase antagonist, exerts its immunosuppressive effects primarily by inhibiting the activation of rapidly dividing cells, including CD4^+^ T helper cells, through the disruption of DNA nucleotide synthesis [33]. This inhibition was modeled via the inhibition of the activation of CD4^+^ T helper cells, as shown below in Equation (11), which replaces Equation (7) in the model. A half-maximal inhibitory concentration for methotrexate (IC50_MTX_) of 1 nM for methotrexate inhibition on AT_hlp_ cell activation was used [34]. The concentration of methotrexate (C_MTX_) was derived from the pharmacokinetic equation governing the plasma concentration of a drug administered via multiple extravascular doses. The detailed equation can be found in the Supplementary Material.

(11) $$\frac{d{AT}_{hlp}}{dt}=\;\delta_{{NT}_{hlp}}\cdot D\cdot {NT}_{hlp}\cdot \left[\frac{{IC50}_{MTX}}{{(C}_{MTX}+{IC50}_{MTX})\;}\right]+\rho_{{AT}_{hlp}}\cdot {AT}_{hlp}\cdot D\cdot \;\left[\frac{{AT}_{reg}50}{{(AT}_{reg}50+{AT}_{reg})\;}\right]-\;\beta_{{AT}_{hlp}}\cdot {AT}_{hlp}.$$

In contrast, the immunosuppressive effect of tacrolimus is mediated by preventing the transcription of interleukin-2 (IL-2) by binding to the FK506-binding protein (FKBP) and thereby blocking cytokine-driven CD4^+^ T helper cell proliferation. Sirolimus, also a FKBP-binding agent, acts downstream by inhibiting the mammalian target of rapamycin (mTOR), which is crucial for the proliferation of activated CD4^+^ T helper cells [35]. Their combined effect was modeled as the inhibition of the proliferation of activated CD4^+^ T helper cells, as described by Equation (12), which replaces Equation (7) in the model for the affected animals. An IC50 of 0.2 nM for tacrolimus/sirolimus inhibition on AT_hlp_ cell proliferation was used [36]. The concentration of tacrolimus (C_TAC_) was derived from the pharmacokinetic equation governing the plasma concentration of a drug administered via multiple extravascular doses. The detailed equation can be found in the Supplementary Material.

(12) $$\frac{d{AT}_{hlp}}{dt}=\;\delta_{{NT}_{hlp}}\cdot D\cdot {NT}_{hlp}+\rho_{{AT}_{hlp}}\cdot {AT}_{hlp}\cdot D\cdot \left[\frac{{AT}_{reg}50}{{(AT}_{reg}50+{AT}_{reg})\;}\right]\cdot \;\left[\frac{{IC50}_{TAC}}{{(C}_{TAC}+{IC50}_{TAC})\;}\right]–\;\beta_{{AT}_{hlp}}\cdot {AT}_{hlp}.$$

Figure 2 illustrates the full model structure. The other model equations and all model parameters are described in detail in the Supplementary Material.

##### Model Assumptions and Limitations

The semi-mechanistic adaptive immune system model was based on the following assumptions and simplifications of the underlying physiologic processes:

1. Plasma was modeled as the space for immune cells to reside in as a well-stirred approximation, assuming that lymphocyte trafficking between blood and extravascular spaces, such as lymphatic organs, is rapid relative to the rate-limiting steps in mounting an ADA response. Thus, the model did not account for the interactions between immune cells and the mAb that ideally take place in lymphoid organs, such as the spleen, lymph nodes, and bone marrow, where concentrations may not be reflective of plasma levels [16,20].
2. Since DCs are the most efficient antigen-presenting cells, they were chosen to represent all antigen-presenting cells in the model [16].
3. The model did not capture the internalization, intracellular antigen processing, MHC-II peptide loading, or antigen presentation processes by MD cells.
4. Efficient antigen presentation by MD cells leads to the activation and proliferation of both CD4^+^ T helper and T regulatory cells, as well as B cells. Subsequently, B cells differentiate into plasma cells that secrete ADA [16]. However, in the current model, ADA formation was directly linked to the number of AT_hlp_ cells. As in a T cell-dependent immune response, AT_hlp_ cells act as the primary driver of B cell activation [29]. Furthermore, the balance between CD4^+^ T helper cells and T regulatory cells is the rate-limiting step in ADA formation [29]. To simplify this process, a lag time (T_lag_) (as shown in Equation (9)) was incorporated to account for the time required for B cell activation, proliferation, and differentiation into plasma cells.
5. No immunomodulatory cytokines, such as IL-2, were included in the model [20].
6. Since ADA assessment is semi-quantitative, it generally does not provide sufficient information about the presence of a memory immune response. So, a memory immune response could not be captured with the observed ADA S/N data. Therefore, memory CD4^+^ T helper or memory T regulatory cells were not incorporated into the model.
7. In the absence of the availability of some rat-specific parameter values, such as death rate constants for ND, MD, NT_hlp_, NT_reg_, and AT_reg_ cells (symbolized as β), the maximum activation rate for ND, NT_hlp_, and NT_reg_ cells (symbolized as δ), and the erenumab concentration at which the ND cell activation rate is at the 50% maximum (symbolized as EC50), these were approximated by using the corresponding values in mice as a closely related rodent species, as previously reported in the literature [16].

##### Statistical Analysis

To evaluate cellular dynamics in the presence or absence of ADA, as simulated by the model, a mixed-effect model was applied to the T cell population at each time point during the induction and rechallenge phases. The analysis aimed to determine if there was a significant increase in the AT_hlp_ cell number compared to the AT_reg_ cell number in the presence or absence of an ADA response. Statistical analyses were performed with Prism software (version 10.0.2, GraphPad Software, Boston, MA, USA).

##### Sensitivity Analysis

A sensitivity analysis was conducted to evaluate the impact of individual parameter values on the state variable ADA magnitude [16]. Control coefficients were used as indicators for sensitivity. The control coefficients of variable x to parameter p (${CC}_{p}^{x}$) were calculated in the following manner [37]:

(13) $${CC}_{p}^{x}=\;\frac{p}{x}\cdot \frac{\partial x}{\partial p},$$

and the computational approximation to the above equation was formed using the finite difference method, as shown below:

(14) $${CC}_{p}^{x}=\;\frac{p}{x(p)}\cdot \frac{x\;\left(p+\;∆p\right)-x\;\left(p\right)}{∆p}.$$

The element Δp is a 1% increase in the value of *p*, which has been shown to be the most numerically stable quantity of variation for this type of sensitivity calculation [16,37]. We reported CC_max_, the ${CC}_{p}^{x}$ value with the maximum absolute value over the time course of the study. Briefly, the larger the value of the control coefficient is, the more sensitive the state variable is to the parameter. A positive CC_max_ indicates that the increase in the parameter value results in an increase in the value of x. Conversely, a negative CC_max_ indicates the parameter value causes a decrease in the x value.

### 2.3. Modeling Software and Parameter Estimation

The dynamic PK/ADA processes were described in terms of linear ordinary differential equations (ODEs) and are listed in detail in the Supplementary Materials. The ODEs describing the serum concentration–time profile of erenumab for the study population in the population PK model were written in the MlxTran format and fitted to the data using the stochastic approximation expectation maximization (SAEM) algorithm of the Monolix Suite software (version 2024R4, Lixoft, Antony, France). The ODEs describing the semi-mechanistic model were initially simulated using MATLAB software (version R2024a, MathWorks, Natick, MA, USA) for model development and exploration. In the second step, the established model was fitted to the observed ADA data using the Monolix suite software, and the selected parameters were estimated during model fitting. During model qualification, Monolix and RStudio (version 2021.09.0, The R Foundation for Statistical Computing, Vienna, Austria) were used. All plots were generated using Prism software (GraphPad software, version 10.0.2, Boston, MA, USA).

## 3. Results

This work presents a semi-mechanistic modeling framework to describe the time-dependent formation of ADA and its impact on serum exposure towards a stereotypical human monoclonal antibody, erenumab, in rats, thereby integrating the dynamics of underlying immune cell populations as drivers for ADA formation in order to facilitate the characterization of the effect of concomitant immunosuppressive drug regimens on ADA formation and immune tolerance. The overall purpose of the modeling work was to explore the potential for maintaining immune tolerance during chronic toxicology studies in rodents, which are typically performed during drug development.

**Figure 3:**
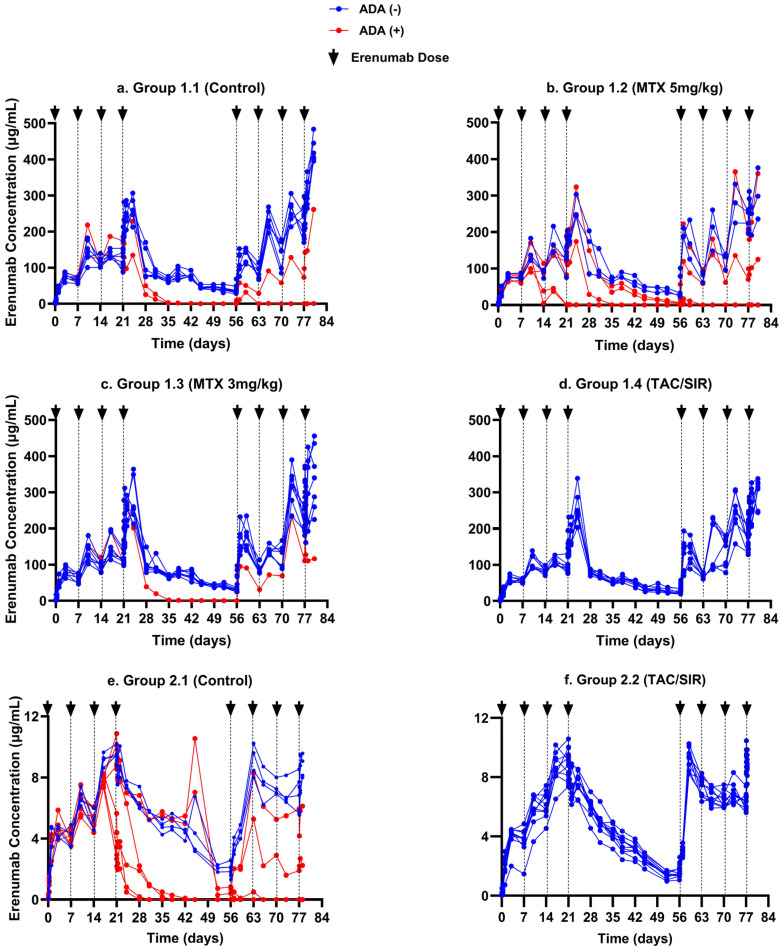
Erenumab serum concentration–time profiles in rats after weekly subcutaneous doses of erenumab in the induction (weeks 1–4) and rechallenge phase (weeks 9–12). The arrows represent the erenumab injection time points. Profiles are separated by the ADA status of the animals: ADA-positive: red; ADA-negative: blue. (**a**) Group 1.1 (control; 10 mg/kg erenumab monomer) without immunosuppression; (**b**) Group 1.2 (once weekly methotrexate 5 mg/kg with 10 mg/kg erenumab monomer); (**c**) Group 1.3 (one initial 3-day cycle of 3 mg/kg methotrexate with 10 mg/kg erenumab monomer); (**d**) Group 1.4 (tacrolimus/sirolimus combination regimen with 10 mg/kg erenumab monomer); (**e**) Group 2.1 (Control; 1 mg/kg erenumab aggregates); (**f**) Group 2.2 (tacrolimus/sirolimus combination regimen with 1 mg/kg erenumab aggregates). From [25].

**Figure 4:**
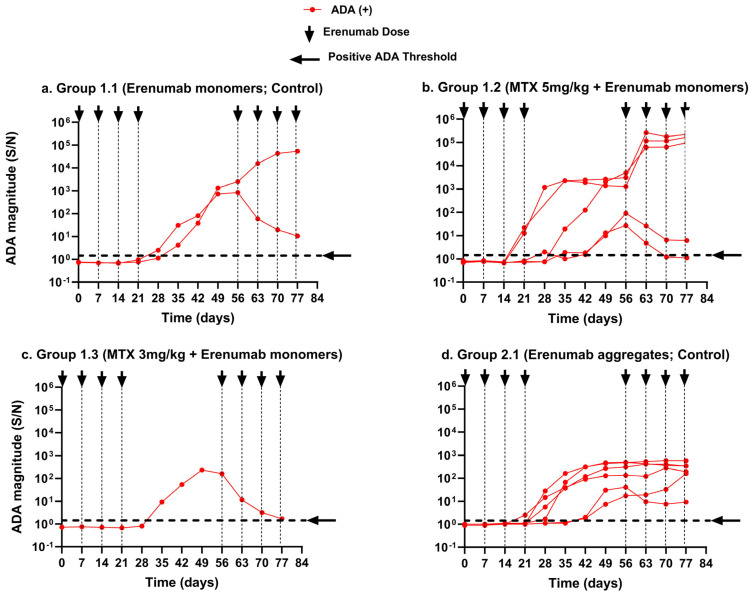
ADA magnitude–time course assessed as a signal-to-noise ratio (S/N) in ADA-positive animals that received erenumab in the induction (weeks 1–4) and rechallenge phases (weeks 9–12): (**a**) Group 1.1 (control; 10 mg/kg erenumab monomer) without immunosuppression; (**b**) Group 1.2 (once weekly methotrexate 5 mg/kg with 10 mg/kg erenumab monomer); (**c**) Group 1.3 (one initial 3-day cycle of 3 mg/kg methotrexate with 10 mg/kg erenumab monomer); (**d**) Group 2.1 (Control; 1 mg/kg erenumab aggregates). The threshold for ADA positivity was set to S/N > 2 for Panels a, b, and c and S/N > 1.5 for Panel d. The arrows represent the erenumab injection time points. The ADA magnitude–time profiles for ADA-negative animals in the above treatment groups were below the ADA-positive threshold and are not displayed. All animals from Group 1.4 (tacrolimus/sirolimus combination regimen with 10 mg/kg erenumab monomer) and Group 2.2 (tacrolimus/sirolimus combination regimen with 1 mg/kg erenumab aggregates) were ADA-negative. Modified from [25].

This modeling analysis was based on two immune tolerance studies previously performed by our group [25], which were designed to characterize ADA formation and erenumab concentration–time profiles over a period of 12 weeks in the presence and absence of methotrexate or a combination of tacrolimus/sirolimus. The study was successfully completed in 52 animals, of which 36 were ADA-negative and 14 were ADA-positive. It provided 1924 erenumab concentrations and 624 ADA data points, collected at various predefined time points, which were used to develop the PK/ADA model. The observed erenumab concentration–time profiles are shown groupwise in Figure 3. The ADA magnitude–time profiles in ADA-positive animals from selected treatment groups in Study 1 and Study 2 are shown in Figure 4.

### 3.1. Population PK Model for Erenumab and Its Modulation by ADA Formation

The structural erenumab PK model was established based on 1924 erenumab serum concentrations obtained from 52 animals in Study 1 and Study 2. The PK of erenumab was best described using a one-compartment model with first-order absorption and first-order elimination from the central compartment. Erenumab absorption kinetics were described using the rate constant Ka, with a mean estimate of 0.91 day^−1^ (7.94% RSE). A large IOV of ~183% (3.94% RSE) was identified for Ka. The value of linear CL/F was estimated to be 1.83 mL/day (3.82% RSE) with an IIV of 17.8% (8.95% RSE). The erenumab central V_d_/F was estimated as 32.6 mL (3.74% RSE). IIV for V_d_/F was defined separately for the two studies: 86.9% (11.6% RSE) for Study 1 and 21.2% (14.3% RSE) for Study 2. The high IIV on V_d_/F in Study 1 is likely attributed to the fact that different dilution protocols were followed during erenumab quantification, as it has been reported that different sample dilutions can lead to variability in the results [38].

The aggregation state of the administered mAb was identified as a significant covariate for CL/F and Ka. Erenumab aggregates led to ~140% (5.60% RSE) higher CL/F and ~88% (13.6% RSE) lower Ka compared to erenumab monomers. This assessment was based on 38 ADA-negative animals and was independent of the impact of ADA on drug clearance.

The ADA S/N ratio was identified as a significant time-varying continuous covariate on CL/F. The ADA effect, quantified by the exponent θ_ADA_, was estimated with precision upon fitting the model to observed ADA S/N data obtained from 14 ADA-positive animals. θ_ADA_ was estimated as 0.37 (3.27% RSE). The positive value of θ_ADA_ suggests a direct relationship between ADA S/N levels and drug clearance, suggesting that as ADA S/N increases, the clearance of the drug also increases. ADA-positive animals exhibited a 3- to 620-fold increase in clearance compared to ADA-negative animals.

Weight was not identified as a covariate influencing CL/F or V_d_/F and, therefore, was not included in the final model.

Residual error was defined using a combined additive and proportional error model. Two separate combined residual error models were used for the two studies. Beal’s M3 method was used to estimate the likelihood that data points were below the limit of quantification (BLQ), as 35% of the erenumab concentration data from 14 ADA-positive animals were below BLQ [39]. All model-based parameters were estimated with satisfactory accuracy, with RSE < 25% and <30% for the fixed effect and random effect parameters, respectively [40] (Table 1).

**Figure 5:**
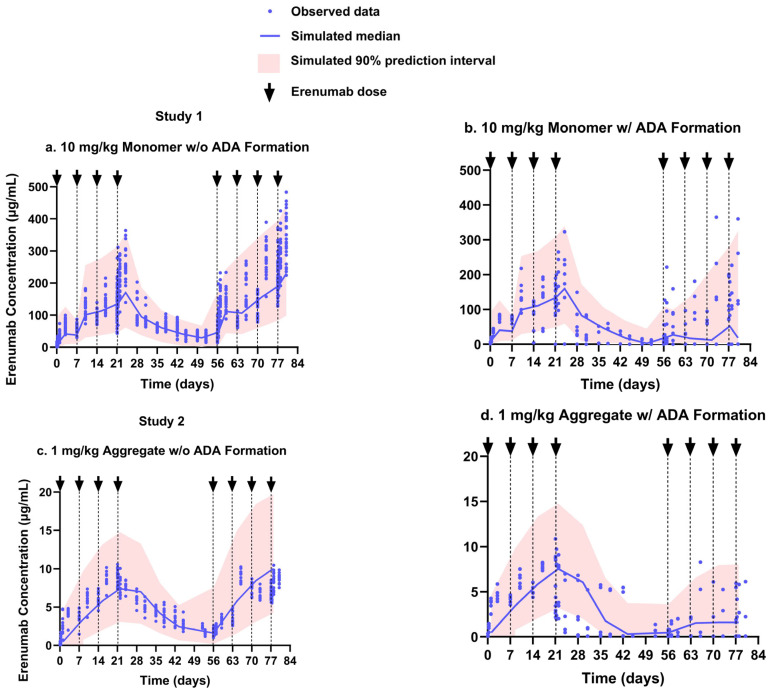
Visual predictive check for the final population pharmacokinetic model: (**a**) 10 mg/kg erenumab monomer dosing without ADA response; (**b**) 10 mg/kg erenumab monomer dosing with ADA response; (**c**) 1 mg/kg erenumab aggregate dosing without ADA response; (**d**) 1 mg/kg erenumab aggregate dosing with ADA response.

The diagnostic goodness-of-fit plots of observed vs. predicted measurements, individual weighted residuals vs. time, and individual weighted residuals vs. individual predictions for the final model suggest that the model described the data adequately (Figure S1, Supplementary Material). Results of a non-parametric bootstrap analysis indicated that all model parameters were estimated with good precision, indicating the stability of the final model (Table 1). The VPC plots for Study 1 indicate that there was no obvious model misspecification (Figure 5a,b). The VPC plot for Study 2 ADA-negative animals suggests that the model slightly overestimated the IIV compared to the variability in the observed data, which is indicated by an elevated shrinkage (ᾑ_shrinkage_) of 42.2% on the apparent V_d_/F for Study 2 and the underlying reason for this discrepancy remains unclear (Figure 5c). The large variability in erenumab concentrations in ADA-positive animals in the VPC suggests that the model only inadequately captured the variability in these animals (Figure 5d). This may be attributed to the fact that the increase in clearance caused by ADA is highly variable between different animals due to the unique and polyclonal nature of each animal’s response to the mAb [41]. In addition, differences in the ADA binding affinity for mAb can also contribute to variability, as similar ADA levels can lead to markedly different effects on the PK of mAb [41].

### 3.2. Semi-Mechanistic Immune Cell Model of Immune Tolerance Induction

The semi-mechanistic model was developed based on 624 ADA samples obtained from 52 animals in Study 1 and Study 2. In the first step of model development, we explored the ability of the model to simulate ADA magnitude–time profiles for animals from the two control and immune-tolerant (methotrexate and tacrolimus/sirolimus) groups. Initial model parameters were obtained directly from studies published by Chen et al. and Hamuro et al. [16,20]. Selected model parameters (EC50_aggregate_, ρ_Athlp_, and β_AThlp_) were calibrated to align the model simulations with the experimentally determined ADA S/N across all treatment groups (Table 2). The higher ADA incidence observed with erenumab aggregates in Group 2.1 was captured by assigning a ~5-fold lower EC50 (EC50_aggregates_: 2.02 μg/mL vs. EC50_monomer_: 9.85 μg/mL) for aggregates compared to the monomer in Group 1.1 (Figure 4a,d). The more potent EC50 for aggregates led to a significant increase in the simulated number of MD cells relative to monomers (Equation (6)), and the model successfully captured the resulting higher ADA incidence rate observed with aggregates. In the subsequent step, selected parameters (ρ_ATreg_, AT_reg_50, and T_lag_) were estimated by fitting the model to the observed ADA S/N for each animal using their respective erenumab doses and PK parameters, as obtained from the final population PK model described above (Table 2). The magnitude of the ADA effect was described in the model by the parameters ρ_ATreg_ and AT_reg_50. ρ_ATreg_ represents the maximum proliferation rate of AT_reg_ cells, and this parameter increased as ADA S/N decreased over time (Figure S2; Supplementary Material). AT_reg_50 is a measurement of the number of AT_reg_ cells required to suppress 50% of AT_hlp_ cells and was directly proportional to the ADA magnitude (Figure S2; Supplementary Material). The parameter estimates for ρ_ATreg_ and AT_reg_50 were dependent on the measured ADA level (S/N). As shown in Figure 4, the magnitude of the ADA response in each ADA-positive animal was different, and this led to IIV of 15.3% and 3.4% for ρ_ATreg_ and AT_reg_50, respectively. The timing of the initiation of the ADA response is represented in the model by T_lag_, which represents the time delay between the activation/proliferation of T cells and the occurrence of ADA. The estimated T_lag_ was approximately 28 days. This is in line with the observed data, where the first ADA rise was measured between days 21 and 42. This variability in the onset time of ADA formation led to a large IIV of 53% on T_lag_. The availability of only ADA S/N data rather than immune cell populations in the study animals limited the determination of point estimates and IIV to these three parameters, as they were rate-limiting and most relevant in determining an ADA response. The remaining parameters involved in the activation, differentiation, and proliferation of immune cells were obtained directly from the literature and fixed in the final model. The full model parameters are described in Table S1 of the Supplementary Material.

In the following, the model performance is separately discussed in four different scenarios.

#### 3.2.1. Scenario 1: Predicted ADA and Simulated Activated CD4^+^ T Helper and Activated T Regulatory Cells in Animals That Received the Erenumab Monomer Alone Without Immunosuppression

**Figure 6:**
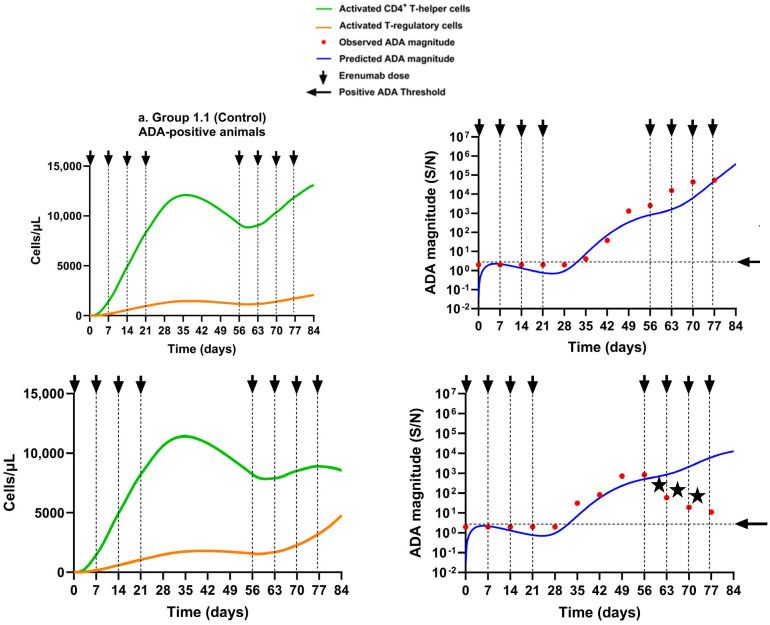

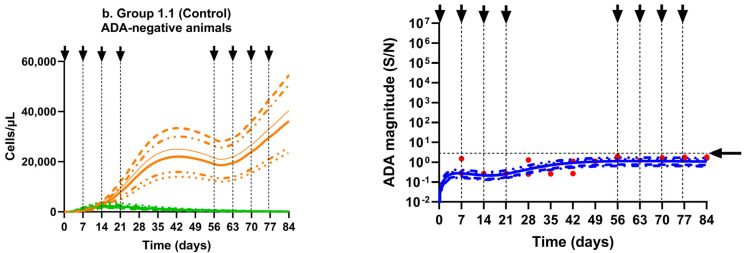
Model simulations for activated CD4^+^ T helper cells and activated T regulatory cells, overlaid with the predicted and observed ADA magnitude–time profiles for the erenumab monomer without immunosuppression. (**a**) Group 1.1: ADA-positive rats; (**b**) Group 1.1: ADA-negative rats. In (**b**), the lines of different styles in the same color are used to distinguish different animals of the same group. Cell numbers are shown on a linear scale, and the ADA magnitude is shown on a logarithmic scale. The positive ADA threshold was set to 2. Arrows represent erenumab dosing times. Stars represent erroneous ADA measurements due to ADA bioanalytical assay limitations.

The semi-mechanistic model adequately described ADA magnitude (S/N) time profiles in the two ADA-positive animals receiving the erenumab monomer alone in Group 1.1 upon model fitting (Figure 6a). The different magnitude of ADA response in the two ADA-positive animals was captured by the model parameters ρ_ATreg_ and AT_reg_50. The parameter T_lag_ adequately captured the onset time of ADA formation in the two animals. The observed decrease in ADA S/N in the second ADA-positive animal at the three time point days 63, 70, and 77 (marked as stars) can be attributed to the fact that weekly dosing with erenumab during the rechallenge phase resulted in drug concentrations exceeding the drug tolerance limit of the ADA bioanalytical assay, probably leading to arbitrarily low ADA measurements. Due to these likely incorrect measurements, ADA S/N at these three time points were excluded from the dataset used for model fitting but were included in the plots. In contrast, the model described the ADA magnitude as being below the ADA positive threshold in the six ADA-negative animals of the group, which correlated well with the observed ADA data (Figure 6b).

To understand the corresponding T cell dynamics, model-simulated profiles of AT_hlp_ and AT_reg_ cells were compared for the animals during the induction and rechallenge phases. The results suggest that the two ADA-positive animals had, on average, seven- to nine-fold higher numbers of AT_hlp_ cells compared to AT_reg_ cells (* *p* < 0.05) during the induction and rechallenge phases, respectively, which resulted in an immune response in these animals (Figure 6a). In contrast, the six ADA-negative animals had, on average, an eight times higher number of AT_reg_ cells than AT_hlp_ cells by the end of the induction phase (* *p* < 0.05), and despite erenumab dosing in the rechallenge phase, this difference increased to ~120 times by the end of the rechallenge phase (** *p* < 0.01), which completely abrogated the ADA response (Figure 6b).

#### 3.2.2. Scenario 2: Predicted ADA and Simulated Activated CD4^+^ T Helper and Activated T Regulatory Cells in Animals That Received the Erenumab Monomer with Immunosuppression

**Figure 7:**
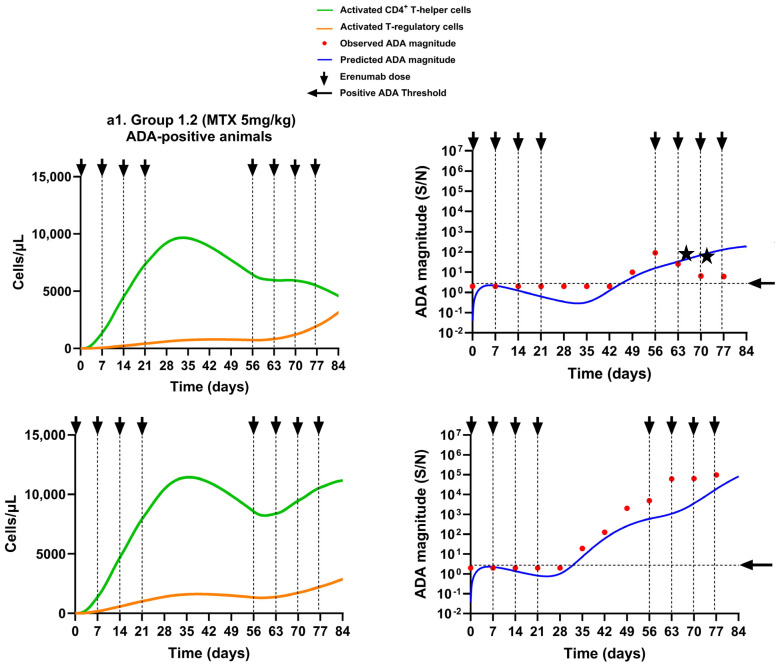

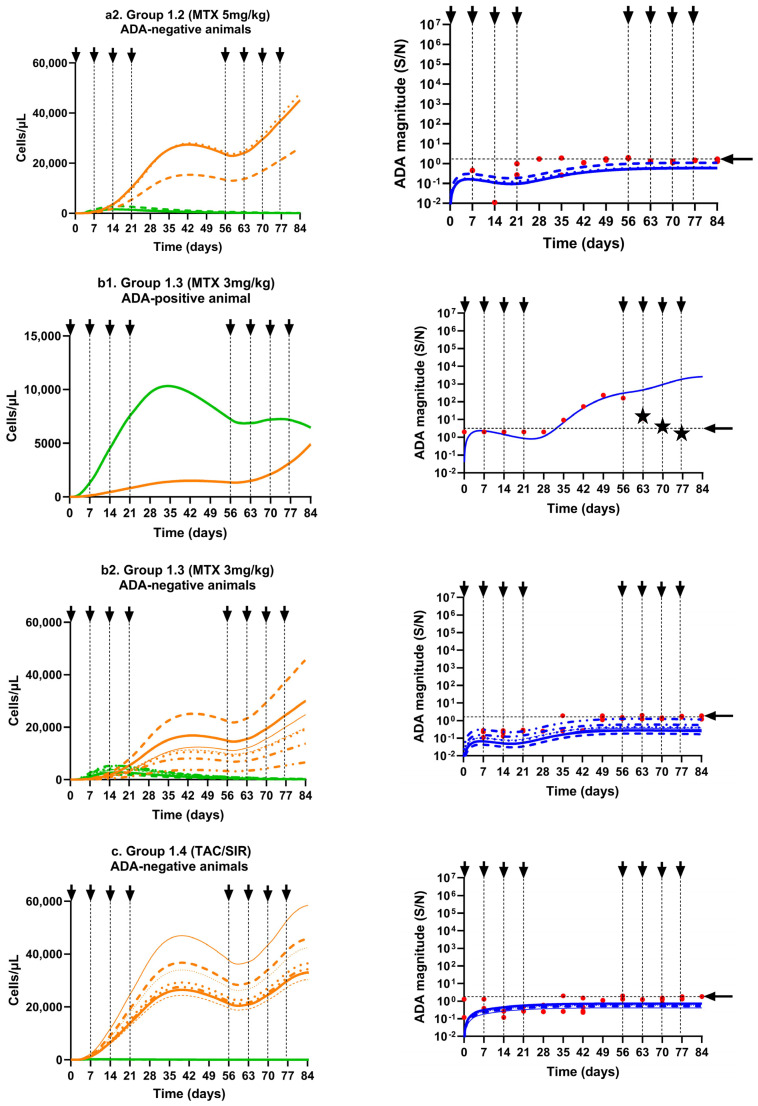
Model simulations for activated CD4^+^ T helper cells and activated T regulatory cells, overlaid with predicted and observed ADA magnitude–time profiles for the erenumab monomer with immunosuppression. (**a1**,**a2**) Group 1.2 animals that received 10 mg/kg of erenumab monomer along with 5 mg/kg methotrexate weekly from week 1 to week 7 with (**a1**) ADA-positive rats and (**a2**) ADA-negative rats. (**b1**,**b2**) Group 1.3 animals that received 10 mg/kg of erenumab monomer along with 3 mg/kg methotrexate on the first, second, and third day with (**b1**) ADA-positive rats and (**b2**) ADA-negative rats. (**c**) Group 1.4 ADA-negative animals that received 10 mg/kg of erenumab monomer along with tacrolimus/sirolimus. In (**a2**,**b2**,**c**), the lines of different styles in the same color are used to distinguish different animals of the same group. Cell numbers are shown on a linear scale, and ADA magnitude is shown on a logarithmic scale. The positive ADA threshold was set to 2. Arrows represent erenumab dosing times. The stars represent erroneous ADA measurements due to ADA bioanalytical assay limitations.

The immunomodulatory action of methotrexate in Groups 1.2 and 1.3 was modeled by inhibiting the activation of CD4^+^ T helper cells, as shown in Equation (11). The model characterized that the Group 1.2 animals, which received weekly doses of methotrexate (5 mg/kg), developed ADA more frequently compared to the Group 1.3 animals that received methotrexate (3 mg/kg) on three consecutive days of week 1 of the study, which correlated well with our observations. The model-derived ADA magnitude–time profiles from selected ADA-positive and ADA-negative animals from the two groups reasonably described the observed ADA magnitude (Figure 7a1–b2).

Upon comparison of the T cell populations, the ADA-positive animals in Group 1.2 had, on average, 92% and 73% more AT_hlp_ cells than AT_reg_ cells by the end of the induction phase and the rechallenge phase, respectively (** *p* < 0.01), and this suggests that continuous dosing with methotrexate did not help to dampen the immune response (Figure 7a1). On the other hand, the ADA-negative animals of the group had approximately 73% and 99% more AT_reg_ cells than AT_hlp_ cells by the end of the induction phase and the rechallenge phase, respectively (** *p* < 0.01), which prevented the ADA response in these animals throughout the study’s duration (Figure 7a2).

The ADA-positive animals in Group 1.3 had, on average, 85% more AT_hlp_ cells than AT_reg_ cells, which resulted in the ADA response (** *p* < 0.01) (Figure 7b1). In contrast, the ADA-negative animals in the group showed a significant decrease in AT_hlp_ cells compared to AT_reg_ cells, with a reduction of approximately 37% at the end of the induction phase and 99% at the end of the rechallenge phase (** *p* < 0.01) (Figure 7b2). This effectively prevented ADA formation in these animals.

To explore the effectiveness of alternate methotrexate regimens, the model was used to simulate whether increasing the methotrexate dose in Group 1.2 could reduce the ADA response in ADA-positive animals from this group. We simulated AT_hlp_ cells, AT_reg_ cells, and ADA S/N profiles for methotrexate doses of 5 mg/kg, 7 mg/kg, and 9 mg/kg, administered once weekly in week 1 to week 7 of the study. The simulated profiles for one representative ADA-positive animal are shown in Figure S3 of the Supplementary Material. The results indicate that higher methotrexate doses do not affect the number of AT_hlp_ or AT_reg_ cells and, consequently, have no impact on the resulting ADA magnitude. We anticipate that a significantly higher dose of methotrexate might alter T cell dynamics and ADA formation, though such a dose could be toxic to rodents. Similar results were observed for the other ADA-positive animals in the group.

Furthermore, the model was utilized to simulate whether adding additional weekly doses of 5 mg/kg methotrexate from week 2 to week 7 to the existing 3 mg/kg methotrexate three-cycle regimen in Group 1.3 could reduce ADA formation in the ADA-positive animal from the group. The simulated profiles for the ADA-positive animal are shown in Figure S4 of the Supplementary Material. The results suggest that increasing the dose and frequency of methotrexate administration had no effect on decreasing the activation of AT_hlp_ cells or ADA magnitude. This suggests that once CD4^+^ T helper cells are activated, the methotrexate regimens explored in this study cannot effectively reduce their activation. Our observations were consistent with previous reports that it is easier to prevent an ADA response than to suppress an existing response since it is more difficult to eliminate activated T cells than naïve T cells [23].

In contrast to methotrexate, the immunosuppressive effect of the tacrolimus/sirolimus combination regimen in Group 1.4 was modeled as the inhibition of AT_hlp_ cell proliferation (Equation (12)). The model-derived ADA magnitude was below the ADA-positive threshold in all animals of the group, which suggests that none of the animals developed ADA, and this is in agreement with the observed ADA data, as shown in Figure 7c. The simulated T cell kinetics suggest that there was a significant increase in AT_reg_ cells compared to AT_hlp_ cells upon tacrolimus/sirolimus treatment. By the end of the induction phase, the AT_reg_ cell numbers were ~10-fold (* *p* < 0.05) higher, and by the end of the rechallenge phase, they were ~1000-fold (*** *p* < 0.001) higher compared to AT_hlp_ cells, which prevented the ADA response in the animals during the induction as well as the rechallenge phase (Figure 7c).

In summary, the model predicts that neither methotrexate regimen was successful in inducing immune tolerance to erenumab. The tacrolimus/sirolimus regimen, however, effectively reduced the activated T helper cell population and induced T regulatory cells, which prevented ADA formation in all the animals.

#### 3.2.3. Scenario 3: Predicted ADA and Simulated Activated CD4^+^ T Helper and Activated T Regulatory Cells in Animals That Received the Erenumab Aggregate Alone Without Immunosuppression

**Figure 8:**
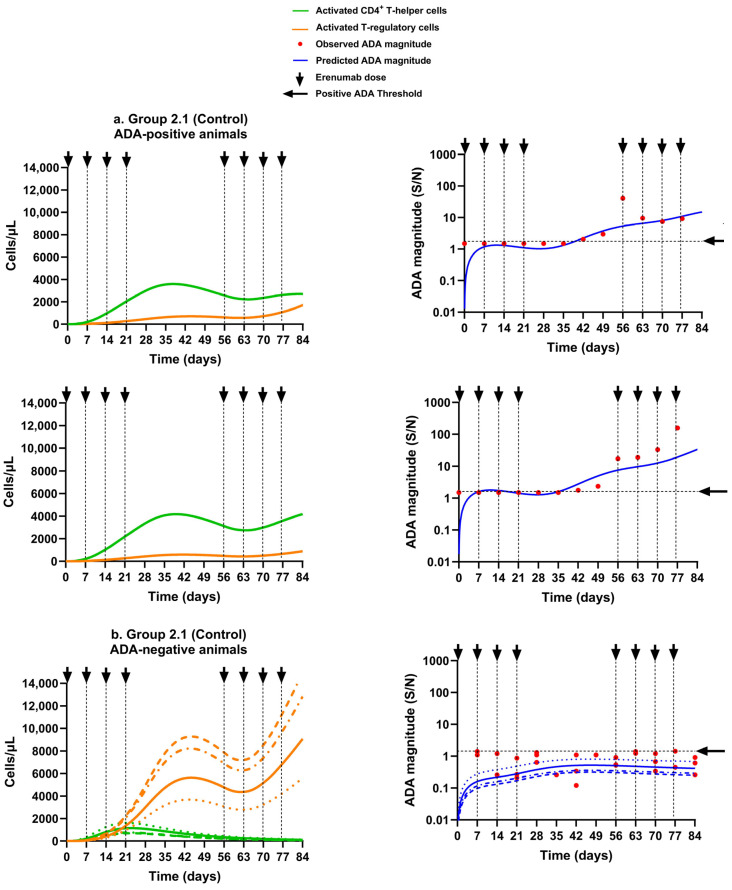
Model simulations for activated CD4^+^ T helper cells and activated T regulatory cells, overlaid with predicted and observed ADA magnitude–time profiles for erenumab aggregate without immunosuppression. (**a**) Group 2.1: ADA-positive rats; (**b**) Group 2.1: ADA-negative rats. In (**b**), the lines of different styles in the same color are used to distinguish different animals of the same group. Cell numbers are shown on a linear scale, and the ADA magnitude is shown on a logarithmic scale. The positive ADA threshold was set to 1.5. Arrows represent erenumab dosing times.

The model behavior for an enhanced immune response towards aggregated erenumab was achieved by assigning a more potent EC50 for the activation of naïve DCs compared to the erenumab monomer (Table 2) since it has been demonstrated that IgG aggregates induce DC maturation and activation much more effectively than their monomeric counterparts because of the upregulation of DC maturation markers (CD83 and CD86) on their surface and the production of inflammatory cytokines and chemokines such as IL-1β, IL-6, IL-8, TNF-α, IL-12, CXCL-10, and MMP-2 [42]. The model characterized higher ADA incidence with erenumab aggregates in Group 2.1 as compared to the erenumab monomers in Group 1.1, which was in agreement with the observed ADA incidence across the two groups. The results suggest that six out of ten animals treated with erenumab aggregates alone developed an ADA response, which aligned with the observed ADA incidence in Group 2.1. In addition, the model-simulated ADA magnitude–time profiles reasonably matched the observed ADA data in the two representative ADA-positive animals (Figure 8a) and four ADA-negative animals (Figure 8b) throughout the study period. On average, the ADA-positive animals exhibited 95% and 97% more AT_hlp_ cells than AT_reg_ cells by the end of the induction and rechallenge phases, respectively (** *p* < 0.01), which resulted in the ADA response (Figure 8a). In contrast, the ADA-negative animals had approximately 10 (* *p* < 0.05) and 100 times (** *p* < 0.01) more AT_reg_ cells than AT_hlp_ cells by the end of the induction and rechallenge phases, respectively, which prevented ADA production in these animals (Figure 8b).

#### 3.2.4. Scenario 4: Predicted ADA and Simulated Activated CD4^+^ T Helper and Activated T Regulatory Cells in Animals That Received the Erenumab Aggregates with Immunosuppression

**Figure 9:**
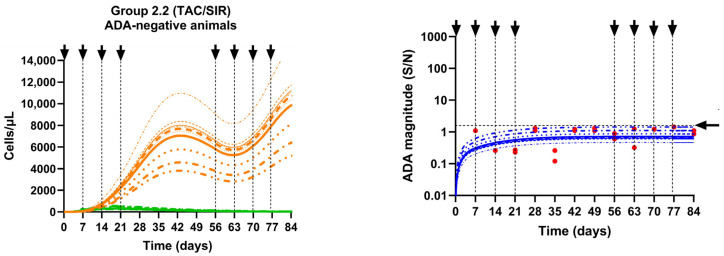
Model simulations for activated CD4^+^ T helper cells and activated T regulatory cells, overlaid with predicted and observed ADA magnitude–time profiles for erenumab aggregate with immunosuppression (Group 2.2). The lines of different styles in the same color are used to distinguish different animals of the same group. Cell numbers are shown on a linear scale, and the ADA magnitude is shown on a logarithmic scale. The positive ADA threshold was set to 1.5. Arrows represent erenumab dosing times.

The model demonstrated that co-administration of erenumab aggregates with a tacrolimus/sirolimus regimen in Group 2.2 prevented ADA in all treated animals. The model simulated ADA magnitude–time profiles and described the observed data adequately (Figure 9). In addition, the simulated profiles of T cells suggest that tacrolimus and sirolimus adequately inhibited the proliferation of AT_hlp_ cells, which led to a significant reduction in their numbers compared to AT_reg_ cells by about 95% and 99% at the end of the induction and rechallenge phase, respectively (** *p* < 0.01), which prevented the ADA response in these animals (Figure 9).

Overall, the results in these different scenarios suggest that the developed semi-mechanistic immune cell model adequately captured the ADA response in animals with and without various immunomodulatory regimens. In addition, the model simulated dynamic profiles of T helper and T regulatory cell populations in ADA-positive and ADA-negative animals across the various treatment groups, which correlated well with the known immunological mechanisms of ADA formation and its pharmacotherapeutic modulation.

#### 3.2.5. Sensitivity Analysis

A sensitivity analysis was performed to evaluate the sensitivity of model parameters and their underlying assumptions for their effect on ADA kinetics, as simulated by the semi-mechanistic immune cell model (Table 3). The sensitivity analysis indicated that the initial number of naïve CD4^+^ T helper cells, including their activation, proliferation, and elimination of CD4^+^ T helper cells, are the most critical processes in the development of ADA. In addition, the initial number of naïve T regulatory cells and the proliferation of activated T regulatory cells are also sensitive model parameters. Particularly, the proliferation rate of activated CD4^+^ T helper cells and activated T regulatory cells has a high impact on the model outcome. This is reasonable when we consider that the number of AT_hlp_ cells relative to AT_reg_ cells has a direct impact on ADA formation. To further increase model credibility, it would be advisable to further confirm these parameter values through in vitro or in vivo experiments in the future. A visual representation of the sensitivity analysis results is shown in Figure S2 in the Supplementary Material.

### 3.3. Model Application for Immune Tolerance Prediction

The final semi-mechanistic model of immune tolerance induction was utilized to predict the maintenance of immune tolerance in a prospective 6-month chronic multiple-dose toxicology study in animals initially tolerized with the tacrolimus/sirolimus regimen. This simulation exercise was intended to explore, within the uncertainties and limitations of our current model, how long immune tolerance persists if the antigen treatment is continued for 6 months of the rechallenge phase without any further dose of tacrolimus or sirolimus.

We simulated AT_hlp_, AT_reg_, and ADA magnitude–time profiles in 1000 animals. A random number generator function was used to sample 1000 ρ_ATreg_ values from a normal distribution, with the mean and standard deviation of the distribution of random effects as estimated in the final model to recreate the random inter-individual variability during simulation. The fixed and random effects for AT_reg_50 were not incorporated because the complexity of the simulations increases with more than one random effect parameter. It also becomes important to understand potential correlations among these parameters and account for the correlations during simulation so as to avoid implausible combinations of parameters in individual subjects. In addition, the sensitivity analysis discussed above suggests that ρ_ATreg_ was a more sensitive parameter than AT_reg_50; hence, it was more important to take into account the fixed and random effects associated with ρ_ATreg_ rather than AT_reg_50.

**Figure 10:**
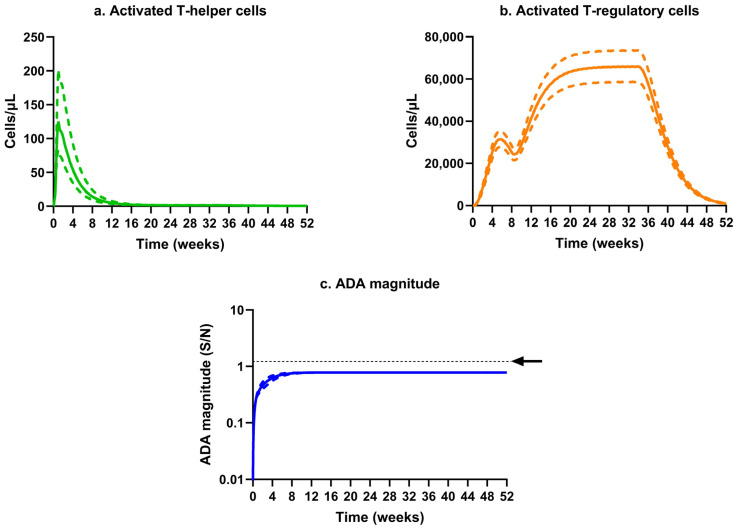
Stochastic simulation of the time courses of T cells and ADA magnitude for a hypothetical chronic toxicology study. (**a**) Activated CD4^+^ T helper cells, (**b**) activated T regulatory cells, and (**c**) ADA magnitude for a hypothetical 6-month multiple-dose toxicology study in 1000 animals that received 10 mg/kg of erenumab monomer weekly for 6 months in the rechallenge phase. The solid line represents the average values, and the dashed line represents the 95% prediction interval. Cell numbers are shown on a linear scale, and the ADA magnitude is shown on a logarithmic scale. The color scheme for the lines is consistent with Figure 6, Figure 7, Figure 8 and Figure 9, where time courses of activated CD4^+^ T helper cells are depicted in green, activated T regulatory cells in orange, and ADA magnitude in blue. The positive ADA threshold was set to 2, represented by an arrow in Panel (**c**).

The results of our simulation suggest that after the induction of the initial immune tolerance with tacrolimus/sirolimus, any further dose of erenumab would prolong and maintain the T regulatory cell-mediated tolerance, as each antigen dose increases and maintains the number of AT_reg_ cells significantly higher than the AT_hlp_ cells, which effectively prevents ADA formation in all animals. This abrogation in the ADA response for an extended duration suggests that the successful conductance of a chronic toxicology study of 6 months duration for a human mAb in rats may be possible. The simulated profiles for animals that received erenumab in monomeric form are shown in Figure 10, and the profiles for erenumab in aggregated form are shown in Figure S5 in the Supplementary Material.

## 4. Discussion

With the advent of an increasing number of chronic drug therapies requiring long-term administration of TPs, mitigation strategies against the often-associated ADA formation using pharmacologic immunomodulation have become increasingly relevant in clinical pharmacotherapy [43]. Similar strategies should be equally applicable and have been explored in preclinical drug development programs when humanized or human TPs are administered to preclinical animal species, such as rats, in chronic toxicology studies [2,25]. These approaches aim to induce immune tolerance, allowing for the accurate assessment of preclinical pharmacokinetics and toxicology of therapeutic proteins without ADA interference [2]. In this current work, we used the capabilities of a model-based population pharmacokinetic analysis along with a semi-mechanistic modeling framework for the adaptive immune system to characterize the time course and impact of ADA formation against stereotypical human mAb in rats under different scenarios of immunomodulation and applied this model to explore the persistence of immune tolerance in chronic toxicology studies after the induction of initial immune tolerance by the short-term administration of immunosuppressive drug regimens.

In our developed model, a one-compartment population PK model reasonably captured the erenumab concentration–time profiles in both ADA-positive and ADA-negative animals across the various immunomodulated and control groups. The effect of ADA was adequately characterized as a continuous, time-varying covariate on drug clearance and provided a means to understand the observed variability in erenumab systemic exposure in ADA-positive animals. Similar compartmental PK models incorporating ADA as a covariate have been previously utilized to evaluate the effect of immunogenicity on the PK of TP [28]. The parameter estimates of the final model (Table 1) were compared with published values to assess their physiological relevance and ensure consistency with known biological parameters. The estimated value of the absorption rate constant, Ka ~0.91 day^−1^, was close to what was reported in a clinical study for subcutaneously administered golimumab (0.91 day^−1^) [44].

The model was unable to simultaneously estimate both IOV and IIV on Ka. Given that IOV (~183%) was relatively large, it likely masked any IIV for Ka. Therefore, IIV on Ka was excluded from the final model. The large IOV is likely due to the fact that the absorption of mAb can vary depending on the subcutaneous administration site [4]. mAbs injected subcutaneously are absorbed from the interstitial space into the lymphatic system, and the rate of absorption can be influenced by the local distribution of the lymph nodes, specific lymphatic drainage pathways, and lymph flow rate. These factors can result in variability not only in mAb’s lymphatic residence time but also in the rate of pre-systemic degradation, which has been suggested to be a function of lymphatic residence time [4]. Our results are furthermore consistent with the findings of Kagan et al., where SC absorption of rituximab was found to be different from three different injection sites in rats [45].

The erenumab V_d_/F in our study was 0.17 L/kg, which is in the range of values typically reported for other mAbs such as alemtuzumab (0.16 L/kg) [46], efalizumab (0.13 L/kg) [47], and ustekinumab (0.22 L/kg) [48], as they are mostly confined to the vascular space and well-perfused organs. V_d_/F had a large IIV of 86.9% and 21.2% observed in the two studies, and close to what has been seen in the other studies for sibrotuzumab (20%) [49], infliximab (22%) [50], and alemtuzumab (85%) [46].

The CL/F estimate in the present study was 1.83 mL/day, a value similar to the clearance of rituximab and another human monoclonal antibody, AMG589, in rats, which were reported as 1.70 mL/day and 1.81 mL/day, respectively [26,45]. CL/F had an IIV of 17.8%, a value similar to the 24% reported for matuzumab [51]. Weight was not identified as a covariate on CL/F or V_d_/F. The animals used in the two studies were adults at the time of purchase, weighing ~270–290 g. By the end of the 12-week study period, their weight increased to ~380–410 g, primarily due to an increase in fat mass. As it is well-known that mAbs are primarily cleared through non-specific proteolytic degradation mediated by the reticuloendothelial system and endothelial cells [4], this weight gain based on fat tissue did not significantly affect drug clearance. Further, erenumab is expected to remain largely confined to the vascular space, as it does not bind to any endogenous targets in rats. Its limited distribution to highly perfused tissues or organs is mediated by convective transport within the interstitial space [4]. Consequently, the observed weight gain likely had a minimal impact on the drug’s volume of distribution.

The estimated value of the ADA effect (θ_ADA_) of 0.37 was similar to the reported value of 0.38 for certolizumab pegol, where ADA was also added as a continuous time-varying covariate in the PK model [52]. Aggregates were shown to have significantly slower absorption and faster drug clearance in our analysis compared to the monomeric form. These observations can be attributed to the large size of protein aggregates compared to monomers. The larger protein molecules are more slowly absorbed by the lymphatic system, and uptake is also reduced as bigger molecules are found to be trapped in the extracellular matrix for longer durations, which results in a reduced rate and extent of SC absorption [53]. In addition, the large aggregates are avidly recognized and phagocytized by the reticuloendothelial system and, therefore, are cleared from the blood at an accelerated rate compared to their non-aggregated counterparts [54].

The semi-mechanistic modeling component provides a quantitative framework to understand adaptive immune system responses and downstream ADA production. The model could reasonably capture immunological mechanisms, such as the activation of dendritic cells and the subsequent dendritic cell-mediated CD4^+^ T helper or T regulatory cell (T_reg_) activation and proliferation.

Administration of erenumab monomer alone in Group 1.1 and erenumab aggregate alone in Group 2.1 resulted in 25% and 60% ADA incidence, respectively (Figure 4). The ADA-positive animals in these groups had, on average, 85–97% more AT_hlp_ cells compared to AT_reg_ cells, which resulted in ADA formation (** *p* < 0.01). On the other hand, the remaining animals in these groups developed immune tolerance to erenumab and did not develop ADA. Tolerance to an injected antigen, particularly after frequent exposure, is a well-documented phenomenon [55,56,57,58,59,60]. Tolerance mechanisms are thought of as a form of protection to minimize inflammation-induced damage to organs/tissues after frequent antigen exposures. Immune tolerance could be invoked by several mechanisms that include both central tolerance (eliminates T and B lymphocytes through negative selection in the thymus) or peripheral tolerance that includes deletion or exhaustion of lymphocytes, upregulation of checkpoint receptors that dampen the response (e.g., programmed death-1 receptor), or increase in T_reg_ cells [61,62]. The T_reg_ pathway was chosen to conceptualize tolerance development in the current model because several quantitative T_reg_ models had already been published in the literature [63,64,65]. We adopted a T helper and T_reg_ cross-regulation model of tolerance induction for autoimmune disease and refined it to align with our study design [30]. The mechanistic basis of the model is the fact that T_reg_ cells have an inhibitory effect on activated T helper cell proliferation [20,30]. As a result, the ADA-negative animals of the control groups in our analysis had approximately 70–80% higher numbers of AT_reg_ cells compared to AT_hlp_ cells (** *p* < 0.01).

We were able to model the immunosuppressive effects of methotrexate and tacrolimus/sirolimus, with the outcome that the tacrolimus/sirolimus combination is not only more effective in suppressing ADA formation but also induces prolonged immune tolerance with its short-term administration. Methotrexate is known to inhibit the activation of T helper cells [66]. Despite the T helper cell suppression mediated by methotrexate, 62.5% and 12.5% of animals in Group 1.2 and Group 1.3, respectively, developed ADA (Figure 4). The remaining ADA-negative animals in these two groups exhibited a significantly higher percentage of AT_reg_ cells compared to AT_hlp_ cells, ranging from 70% to 99% (** *p* < 0.01). These results correspond well to the observed increase in Treg activation marker CD4^+^Foxp3^+^ and the absolute number of T_regs_ in rheumatoid arthritis patients treated with methotrexate [67].

The tacrolimus/sirolimus effect was conceptualized by inhibiting the proliferation of activated T helper cells [35]. Inhibition by tacrolimus/sirolimus completely mitigated the ADA response throughout the study, not only against the erenumab monomer in Group 1.4 but also towards the more immunogenic erenumab aggregates in Group 2.2. The model-simulated AT_reg_ profiles suggest a significant increase in their numbers compared to AT_hlp_ cells by ~75–97% (** *p* < 0.01). Previously, tacrolimus was shown to induce tolerance by increasing the proliferation of T_regs_ in liver and kidney transplant patients [68,69]. In summary, these results suggest that the different mechanisms of action employed by these different immunosuppressive drugs contribute to the differences in their effects on ADA formation. This was further supported by results from the sensitivity analysis, which showed that the T helper proliferation rate (ρ_AThlp_) was a more sensitive parameter compared to the T helper activation rate (δ_NThlp_), suggesting that tacrolimus has a more efficient inhibitory effect on CD4^+^ T helper cells than methotrexate (Table 2).

Lastly, our simulation results suggest that the short course of immune tolerance induction with tacrolimus/sirolimus can confer lasting tolerance with continuous erenumab administration. This would allow the conductance of chronic 6-month toxicology studies for TPs such as mAbs without ADA interference in rodents, as required to support their regulatory approval [70]. The underlying mechanism of tolerance induction in our modeling framework was based on the restoration and maintenance of T_reg_ cells (Figure 10). Interestingly, patients with a rare genetic disorder, Pompe disease, developed prolonged tolerance to continuous enzyme replacement therapy for ~2–4.5 years after the initial immunomodulation therapy with methotrexate, rituximab, and/or intravenous immunoglobulin (IVIG) administered over a period of ~24–36 months [71,72,73]. IVIG and methotrexate have been shown to trigger the production of T_regs_ [72,74,75]. In addition, T_reg_-induced tolerance development has also been successfully used in the treatment of chronic allergic, autoimmune, and inflammatory diseases [76,77].

## 5. Conclusions

In conclusion, this work demonstrates the feasibility of using a semi-mechanistic modeling framework that captures the fundamental immune cell biology of dendritic cells, CD4^+^ T helper cells, and T regulatory cells to predict ADA for a human monoclonal antibody in rats. The model was used to predict the dynamics of T helper and T regulatory cells required for ADA production, as well as how ADA impacts the PK of the mAb under different dosing regimens, mAb aggregation states, and concomitant immunosuppressive therapy. Having a model-based framework to simulate ADA allowed us to perform stochastic simulations, suggesting that a long-term toxicology study can be conducted for mAbs in rats with initial short-term immunomodulation using tacrolimus/sirolimus. The model may potentially be further leveraged as a tool to evaluate the impact of different immune-modulating therapies in indications such as organ transplantation, as well as to explore different dosing regimens and study designs on how to minimize ADA impact on PK and efficacy of TPs in preclinical and clinical trials. This modeling strategy also provides the opportunity to be extended to simulate ADA responses to other TPs by integrating their substance-specific pharmacokinetic parameters. It can also be adapted to other species by incorporating species-specific parameters of immune system components.

## Figures and Tables

**Table 1:** Population pharmacokinetic parameter estimates for erenumab in rats, with erenumab aggregation state and anti-drug antibody magnitude as covariates (see Section 3.1 for details).

Parameter	Estimate (% RSE)	IIV (%)	IOV (%)	Non-Parametric Bootstrap Median (90% CI; n = 1000)
Base CL/F, mL/day	1.83 (3.82)	17.8		1.81 (1.72, 1.93)
Base V_d_/F, mL	32.6 (3.74)	86.9 (Study 1) 21.2 (Study 2)		32.4 (28.0, 35.6)
Ka, day^−1^	0.91 (7.94)		183.0	0.94 (0.80, 1.15)
θ_aggregation, CL_	2.40 (5.60)			2.50 (2.24, 2.80)
θ_aggregation, Ka_	0.12 (13.6)			0.11 (0.08, 0.15)
θ_ADA_ Exponent in ADA effect on CL/F	0.37 (3.27)			0.38 (0.32, 0.45)
Additive residual error (Study 1)	0.09 (22.2)			0.1 (0.06, 0.37)
Proportional residual error (Study 1)	0.29 (2.40)			0.28 (0.25, 0.32)
Additive residual error (Study 2)	0.12 (12.4)			0.12 (0.06, 0.18)
Proportional residual error (Study 2)	0.13 (3.45)			0.13 (0.11, 0.16)

IIV: inter-individual variability, IOV: inter-occasion variability, CI: confidence interval, CL/F: clearance, V_d_/F: volume of distribution, Ka: absorption rate constant, and ADA: anti-drug antibody.

**Table 2:** Semi-mechanistic immune cell model parameter estimates. Parameters were obtained from three sources: (A) Model Calibration, (B) Model Fitting, and (C) the Published Literature (see Section 3.2 and the Supplementary Material for further details and the utilized literature sources).

Parameter	Description	Unit	Value (%RSE)	IIV (%)
**A. Model Calibration**				
EC50_aggregate_	Erenumab aggregate concentration at which the naïve dendritic cell activation rate is 50% maximum	μg/mL	2.02	
ρ_AThlp_	Maximum proliferation rate for activated CD4^+^ T helper cells	Day^−1^	2.45	
β_AThlp_	Death rate for activated CD4^+^ T helper cells	Day^−1^	0.05	
**B. Model Fitting**				
ρ_ATreg_	Maximum proliferation rate for activated T regulatory cells	Day^−1^	3.02 (3.01)	15.3
AT_reg_50	Number of activated T regulatory cells required for half-maximal suppression of activated CD4^+^ T helper cells	Cells/μL	1000	3.4
T_lag_	Lag time for ADA formation	Day	28.3 (2.19)	53
**C. Published Literature**				
β_ND_	Death rate for naïve dendritic cells	Day^−1^	0.0924	
ND_0_	Initial number of naïve dendritic cells	Cells/μL	3700	
δ_ND_	Maximum activation rate for naïve dendritic cells	Day^−1^	1.5	
EC50_monomer_	Erenumab monomer concentration at which naïve dendritic cell activation rate is at the 50% maximum	μg/mL	9.85	
β_MD_	Death rate for mature dendritic cells	Day^−1^	0.2310	
NT_hlp,0_	Initial number of naïve CD4^+^ T helper cells	Cells/μL	723	
β_NThlp_	Death rate for naïve CD4^+^ T helper cells	Day^−1^	0.0056	
δ_NThlp_	Maximum activation rate for naïve CD4^+^ T helper cells	Day^−1^	1.5	
NT_reg,0_	Initial number of naïve T regulatory cells	Cells/μL	62	
β_ATreg_	Death rate for activated T regulatory cells	Day^−1^	0.18	
β_NTreg_	Death rate for naïve T regulatory cells	Day^−1^	0.0056	
δ_NTreg_	Maximum activation rate for naïve T regulatory cells	Day^−1^	1.5	
α	Secretion rate of antibodies	nM/day	77	
K_ADA_	Elimination rate of antibodies	Day^−1^	0.138	

**Table 3:** Sensitivity analysis: 10 sensitive parameters were obtained for the variable of interest, anti-drug antibody.

Parameter	Parameter Description	CC_max_
ρ_ATreg_	Maximum proliferation rate for activated T regulatory cells	−85.0
ρ_AThlp_	Maximum proliferation rate for activated CD4^+^ T helper cells	+83.2
NT0_reg_	Initial number of naïve T regulatory cells	−10.2
AT_reg_50	Number of activated T regulatory cells required for half-maximal suppression of activated CD4^+^ T helper cells	+10.1
NT0_hlp_	Initial number of naïve CD4^+^ T helper cells	+10.0
EC50	Erenumab concentration at which naïve dendritic cell activation rate is at the 50% maximum	−9.35
β_AThlp_	Death rate of activated CD4^+^ T helper cells	−5.12
δ_NThlp_	Maximum activation rate for naïve CD4^+^ T helper cells	+2.35
$K_{ADA}$	Elimination rate constant of antibodies	−2.05
α	Secretion rate of antibodies	+2.00

## Data Availability

Data generated in this study are available on request from the corresponding author.

## Supplementary Materials

The following supporting information can be downloaded at https://www.mdpi.com/article/10.3390/pharmaceutics17070845/s1. References [78,79,80,81,82,83,84,85,86,87] are cited in the supplementary materials. Table S1: Model parameters and their definitions; Figure S1: Diagnostic plots for the final population pharmacokinetic model; Figure S2: Sensitivity analysis of the effect of select parameters on ADA magnitude time courses; Figure S3: Simulated times courses of activated T cells and ADA magnitude for different methotrexate regimens; Figure S4: Simulated times courses of activated T cells and ADA magnitude for different methotrexate regimens; Figure S5: Stochastic simulation of the time courses of T cells, ADA magnitude for a hypothetical chronic toxicology study with erenumab aggregate.
