# Supplementary material for: A Semi-Mechanistic Mathematical Model of Immune Tolerance Induction to Support Preclinical Studies of Human Monoclonal Antibodies in Rats
Source: Pharmaceutics. 2025 Jun 27;17(7):845. doi: 10.3390/pharmaceutics17070845 (PMC12300661; doi:10.3390/pharmaceutics17070845)
Supplement: Supplementary file 1 [file pharmaceutics-17-00845-s001.zip › pharmaceutics-3651277-supplementary.pdf]

Pharmaceutics

A Semi-Mechanistic Mathematical Model of Immune Tolerance Induction to Support Preclinical Studies of Human Monoclonal Antibodies in Rats

Paridhi Gupta, Josiah T. Ryman, Vibha Jawa, and Bernd Meibohm

Blood Sampling Strategy for Study 1 and Study 2 and Sample Handling

Serum samples were collected from all animals at predefined time points for ADA and erenumab quantification. For erenumab PK, predose and 72-hour post-dose samples were obtained weekly from week 1 to week 12, with additional samples at 2, 8, and 24 hours post-dose in weeks 1, 4, 9, and 12. ADA samples were collected at pre-dose every week from week 1 to week 12. Blood samples (0.3 mL) were obtained from tail vein or saphenous vein under isoflurane anesthesia and placed into non-heparinized microtainer tubes (Thermo Fisher Scientific, Waltham, MA). Blood was allowed to clot at room temperature for 30–45 minutes, and serum was separated by centrifugation at 2000 g for 15 minutes at 4°C. All serum samples were stored at -70°C until analysis.

Bioanalytical Assay for ADA Quantification

An acid-dissociation bridging electrochemiluminescence immunoassay (ECLIA) was utilized to measure the relative amount of ADA in the rat serum samples [41,78]. Serum samples were diluted 1:10 with 300mM acetic acid to dissociate any ADA-drug complex. The acidified samples were then incubated in an assay buffer (0.5% BSA, 0.05% Tween20, 0.25% CHAPS, 5 mM EDTA and 0.35 M sodium chloride in PBS) containing biotin- and sulfo-labelled erenumab followed by neutralized with 1 M Tris HCl (pH 8.5) on a streptavidin-coated MSD 600 (Meso® QuickPlex SQ 120 MM, Gaithersburg, MD) plate for 2 hours, followed by washing and analysis on a SECTOR Imager 600 instrument (MSD, Gaithersburg, MD). The response was measured as electrochemiluminescence (ECL) units. ECL signals were expressed as signal-to-noise ratio (S/N), comparing post-dose sample signal to pre-dose sample signal. S/N > 2 (Study 1) and S/N > 1.5 (Study 2) indicated positive ADA reaction. Donkey anti-human IgG antibody (lot 153215, Jackson ImmunoResearch, WestGrove, PA) at following concentrations 0, 10, 100 and 1000 ng/mL were included on every plate as quality controls. The assay could tolerate 40 μg/mL of excess erenumab in the presence of 100 ng/mL of the donkey anti-human IgG antibody.

Bioanalytical Assay for Erenumab Quantification

An ECLIA-based assay was used to quantify erenumab concentrations in rat serum samples [2]. Streptavidin-coated 96-well plates (Meso QuickPlex SQ 120 MM, Gaithersburg, MD) were coated with a biotin-tagged mouse antihuman IgG (lot G3520-ZK500, cat no 9040-08, Southern Biotech, Birmingham, AL) against the heavy chain constant region of erenumab. Standards and quality controls were prepared by spiking erenumab into rat serum and diluted 1:20 in the assay buffer before loading onto the plate. A sulfo-tagged goat anti-human IgG (lot W0019109S, cat no D20QG-6, MSD, Gaithersburg, MD) against the human lambda light chain constant region of erenumab was used for detection. After washing, a tripropylamine read buffer (MSD, Gaithersburg, MD) was added and the plate was then read using a SECTOR Imager 600 instrument (MSD, Gaithersburg, MD). The resulting signal was reported in ECL units. The range of this bioanalytical method was 0.078–10 μg/mL.

Ordinary Differential Equations for the Final Model

Equation S1: Dosing compartment (Dose, unit: μg). The equation terms are: (1) initial dose of erenumab injected; (2) absorption of erenumab

$$\frac{dDose}{dt} = In_{dose}(t) - Ka \cdot Dose$$

Equation S2: Concentration of erenumab in central compartment (A, unit: μg). The equation terms are: (1) amount of erenumab absorbed; (2) elimination of erenumab

$$\frac{dC}{dt} = \frac{Ka \cdot Dose}{V_d/F} - \frac{CL/F}{V_d/F} \cdot C$$

Equation S3: Maturation signal (MS, unit: μg/mL). The equation terms are: (1) concentration of erenumab in the central compartment

$$MS = C$$

Equation S4: Naïve dendritic cells (ND, unit: cells/μL). The equation terms are: (1) formation of naïve dendritic cells based on homeostasis assumption; (2) natural death; (3) activation via encountering MS (concentration of erenumab), modeled using Michaelis–Menten kinetics driven by MS.

$$\frac{\mathbf{dND}}{\mathbf{dt}} = \boldsymbol{\beta}_{\mathbf{ND}} \cdot \mathbf{ND_0} - \boldsymbol{\beta}_{\mathbf{ND}} \cdot \mathbf{ND} - \left[\frac{\boldsymbol{\delta}_{\mathbf{ND}} \cdot \mathbf{C}}{\mathbf{EC50 + C}}\right] \cdot \mathbf{ND}$$

Equation S5: Mature dendritic cells (MD, unit: cells/μL). The equation terms are: (1) formation of MD from ND activation; (2) natural death

$$\frac{\mathbf{dMD}}{\mathbf{dt}} = \left[\frac{\boldsymbol{\delta}_{\mathbf{ND}} \cdot \mathbf{C}}{\mathbf{EC50 + C}}\right] \cdot \mathbf{ND} - \boldsymbol{\beta}_{\mathbf{MD}} \cdot \mathbf{MD}$$

Equation S6: Naïve CD4+ T-helper cells (NT_{hlp}, unit: cells/μL). The equation terms are: (1) formation of naïve T-helper cells based on homeostasis assumption; (2) natural death; (3) activation of NT_{hlp} cells via interaction with MD cells. D is function of interaction between NT_{hlp} cells and MD cells.

$$\frac{\mathbf{dNT_{hlp}}}{\mathbf{dt}} = \boldsymbol{\beta}_{\mathbf{NT_{hlp}}} \cdot \mathbf{NT_{hlp,0}} - \boldsymbol{\beta}_{\mathbf{NT_{hlp}}} \cdot \mathbf{NT_{hlp}} - \boldsymbol{\delta}_{\mathbf{NT_{hlp}}} \cdot \mathbf{D} \cdot \mathbf{NT_{hlp}}$$

$$\mathbf{D} = \frac{\mathbf{MD}}{\mathbf{MD + NT_{hlp} + AT_{hlp} + NT_{reg} + AT_{reg}}}$$

Equation S7: Activated CD4+ T-helper cells (AT_{hlp}, unit: cells/μL). The equation terms are: (1) differentiation of AT_{hlp} cells from NT_{hlp} cells; (2) proliferation of AT_{hlp} cells and inhibition of AT_{hlp} cell proliferation by AT_{reg} cells; (3) natural death

$$\frac{\mathbf{dAT_{hlp}}}{\mathbf{dt}} = \boldsymbol{\delta}_{\mathbf{NT_{hlp}}} \cdot \mathbf{D} \cdot \mathbf{NT_{hlp}} + \boldsymbol{\rho}_{\mathbf{AT_{hlp}}} \cdot \mathbf{AT_{hlp}} \cdot \mathbf{D} \cdot \left[\frac{\mathbf{AT_{reg}50}}{\mathbf{(AT_{reg}50 + AT_{reg})}}\right] - \boldsymbol{\beta}_{\mathbf{AT_{hlp}}} \cdot \mathbf{AT_{hlp}}$$

Equation S8: Naïve T-regulatory cells (NT_{reg}, unit: cells/μL). The equation terms are: (1) formation of naïve T-regulatory cells based on homeostasis assumption; (2) natural death; (3) activation of NT_{reg} cells via interaction with MD cells. D is also the function of interaction between NT_{reg} cells and MD cells.

$$\frac{\mathbf{dNT_{reg}}}{\mathbf{dt}} = \boldsymbol{\beta}_{\mathbf{NT_{reg}}} \cdot \mathbf{NT_{reg,0}} - \boldsymbol{\beta}_{\mathbf{NT_{reg}}} \cdot \mathbf{NT_{reg}} - \boldsymbol{\delta}_{\mathbf{NT_{reg}}} \cdot \mathbf{D} \cdot \mathbf{NT_{reg}}$$

Equation S9: Activated T-regulatory cells (AT_{reg}, unit: cells/μL). The equation terms are: (1) differentiation of AT_{reg} cells from NT_{reg} cells; (2) proliferation of AT_{reg} cells; (3) natural death

$$\frac{\mathbf{dAT_{reg}}}{\mathbf{dt}} = \boldsymbol{\delta}_{\mathbf{NT_{reg}}} \cdot \mathbf{D} \cdot \mathbf{NT_{reg}} + \boldsymbol{\rho}_{\mathbf{AT_{reg}}} \cdot \mathbf{AT_{reg}} \cdot \mathbf{D} - \boldsymbol{\beta}_{\mathbf{AT_{reg}}} \cdot \mathbf{AT_{reg}}$$

Equation S10: ADA formation (ADA, unit: nmol/μL). The equation terms are: (1) formation of ADA via AT_{hlp} cells

$$\mathbf{ADA} = \boldsymbol{\alpha} \cdot \frac{\mathbf{AT_{hlp}}}{\mathbf{K}} \cdot \left[\mathbf{1} - \mathbf{e}^{(-\mathbf{K_{ADA}} \cdot (\mathbf{t} - \mathbf{T_{lag}}))}\right] \text{ for } \mathbf{t} \geq \mathbf{T_{lag}} \text{ and } \mathbf{ADA} = \mathbf{0} \text{ for } \mathbf{t} < \mathbf{T_{lag}}$$

Equation S11: ADA magnitude (S/N) (ADA_{mag}, unit: dimensionless).

$$\mathbf{ADA_{mag}} = \frac{\mathbf{ADA}}{\mathbf{N}}$$

Equation S12: Tacrolimus plasma concentration (C_{TAC}, unit: mg/L).

$$C_{TAC} = \frac{F_{TAC} \cdot D_{TAC} \cdot Ka_{TAC}}{V_{d_{TAC}} \cdot (Ka_{TAC} - Ke_{TAC})} \cdot \left[\frac{e^{-Ke_{TAC} \cdot t}}{1 - e^{-Ke_{TAC} \cdot \tau_{TAC}}} - \frac{e^{-Ka_{TAC} \cdot t}}{1 - e^{-Ka_{TAC} \cdot \tau_{TAC}}}\right]$$

Equation S13: Impact of tacrolimus on the proliferation of activated CD4+ T-helper cells in animals treated with combination tacrolimus and sirolimus in Group 1.4 of Study 1 and Group 2.2 of Study 2.

$$\frac{\mathbf{dAT_{hlp}}}{\mathbf{dt}} = \boldsymbol{\delta}_{\mathbf{NT_{hlp}}} \cdot \mathbf{D} \cdot \mathbf{NT_{hlp}} + \boldsymbol{\rho}_{\mathbf{AT_{hlp}}} \cdot \mathbf{AT_{hlp}} \cdot \mathbf{D} \cdot \left[\frac{\mathbf{AT_{reg}50}}{(\mathbf{AT_{reg}50} + \mathbf{AT_{reg}})}\right] \cdot \left[\frac{\mathbf{IC50_{TAC}}}{(\mathbf{C_{TAC}} + \mathbf{IC50_{TAC}})}\right] - \boldsymbol{\beta}_{\mathbf{AT_{hlp}}} \cdot \mathbf{AT_{hlp}}$$

Equation S14: Methotrexate plasma concentration (C_{MTX}, unit: mg/L).

$$C_{MTX} = \frac{F_{MTX} \cdot D_{MTX} \cdot Ka_{MTX}}{V_{d_{MTX}} \cdot (Ka_{MTX} - Ke_{MTX})} \cdot \left[\frac{e^{-Ke_{MTX} \cdot t}}{1 - e^{-Ke_{MTX} \cdot \tau_{MTX}}} - \frac{e^{-Ka_{MTX} \cdot t}}{1 - e^{-Ka_{MTX} \cdot \tau_{MTX}}}\right]$$

Equation S15: Impact of methotrexate on the activation of CD4+ T-helper cells in animals treated with methotrexate in Groups 1.2 and 1.3 of Study 1.

$$\frac{\mathbf{dAT_{hlp}}}{\mathbf{dt}} = \boldsymbol{\delta}_{\mathbf{NT_{hlp}}} \cdot \mathbf{D} \cdot \mathbf{NT_{hlp}} \cdot \left[\frac{\mathbf{IC50_{MTX}}}{(\mathbf{C_{MTX}} + \mathbf{IC50_{MTX}})}\right] + \boldsymbol{\rho}_{\mathbf{AT_{hlp}}} \cdot \mathbf{AT_{hlp}} \cdot \mathbf{D} \cdot \left[\frac{\mathbf{AT_{reg}50}}{(\mathbf{AT_{reg}50} + \mathbf{AT_{reg}})}\right] - \boldsymbol{\beta}_{\mathbf{AT_{hlp}}} \cdot \mathbf{AT_{hlp}}$$

Table S1. Model parameters and their definitions

Parameter	Description	Unit	Value (%RSE)	Reference
In_{dose}	Erenumab dose	μg		
Ka	Absorption rate constant	Day^{-1}	*	
CL	Clearance	mL/day	*	
V_d	Central volume of distribution	mL	*	
β_{ND}	Death rate for naïve dendritic cells	Day^{-1}	0.0924	[79]
ND0	Initial number of naïve dendritic cells	Cells/μL	3700	Flow cytometry
δ_{ND}	Maximum activation rate for naïve dendritic cells	Day^{-1}	1.5	[80]
$EC50_{monomer}$	Erenumab monomer concentration at which naïve dendritic cell activation rate is 50% maximum	μg/mL	9.85	[81]
$EC50_{aggregate}$	Erenumab aggregate concentration at which naïve dendritic cell activation rate is 50% maximum	μg/mL	2.02	Model calibration
β_{MD}	Death rate for mature dendritic cells	Day^{-1}	0.2310	[82]
$NT0_{hlp}$	Initial number of naïve CD4+ T-helper cells	Cells/μL	723	Flow cytometry
β_{NThlp}	Death rate for naïve CD4+ T-helper cells	Day^{-1}	0.0056	[83]
δ_{NThlp}	Maximum activation rate for naïve CD4+ T-helper cells	Day^{-1}	1.5	[80]
ϱ_{AThlp}	Maximum proliferation rate for activated CD4+ T-helpers	Day^{-1}	2.45	Model calibration
β_{AThlp}	Death rate for activated CD4+ T-helper cells	Day^{-1}	0.05	Model calibration
$NT0_{reg}$	Initial number of naïve T-regulatory cells	Cells/μL	62	Flow cytometry
ϱ_{ATreg}	Maximum proliferation rate for activated T-regulatory	Day^{-1}	3.02 (3.01)	Model fitting
β_{NTreg}	Death rate for naïve T-regulatory cells	Day^{-1}	0.0056	[83]
δ_{NTreg}	Maximum activation rate for naïve T-regulatory cells	Day^{-1}	1.5	[80]
β_{ATreg}	Death rate for activated T-regulatory cells	Day^{-1}	0.18	[84]
$AT_{reg}50$	Number of activated T-regulatory cells required for half-maximal suppression of activated CD4+ T-helper cells	Cells/μL	1000	Model fitting
α	Secretion rate of antibody	nmol/day	77	[26]
T_{lag}	Lag time for ADA formation	Day	28.3 (2.19)	Model fitting
K_{ADA}	Elimination rate of antibody	Day^{-1}	0.138	[85]
K	Constant	Cells/day	500	Model calibration
N	Constant	nmol/μL	1000	Model calibration
$IC50_{TAC}$	Half-maximal inhibitory concentration for tacrolimus	nM	0.2	[36]
F_{TAC}	Bioavailability	Dimensionless	0.2	[36]
D_{TAC}	Dose	mg/kg	2	
τ_{TAC}	Dosing interval	Day	7	
Ka_{TAC}	Absorption rate constant	Day^{-1}	7.2	[36]
Ke_{TAC}	Elimination rate constant	Day^{-1}	3.6	[86]
Vd_{TAC}	Volume of distribution	L/kg	10	[86]
$IC50_{MTX}$	Half-maximal inhibitory concentration for methotrexate	nM	1	[34]
F_{MTX}	Bioavailability	Dimensionless	1	[87]
D_{MTX}	Dose	mg/kg	5 or 3	
τ_{MTX}	Dosing interval	Day	7 or 1	
Ka_{MTX}	Absorption rate constant	Day^{-1}	88.56	[87]
Ke_{MTX}	Elimination rate constant	Day^{-1}	56.41	[87]
Vd_{MTX}	Volume of distribution	L/kg	0.174	[87]

* Obtained from population pharmacokinetic modeling for each individual animal

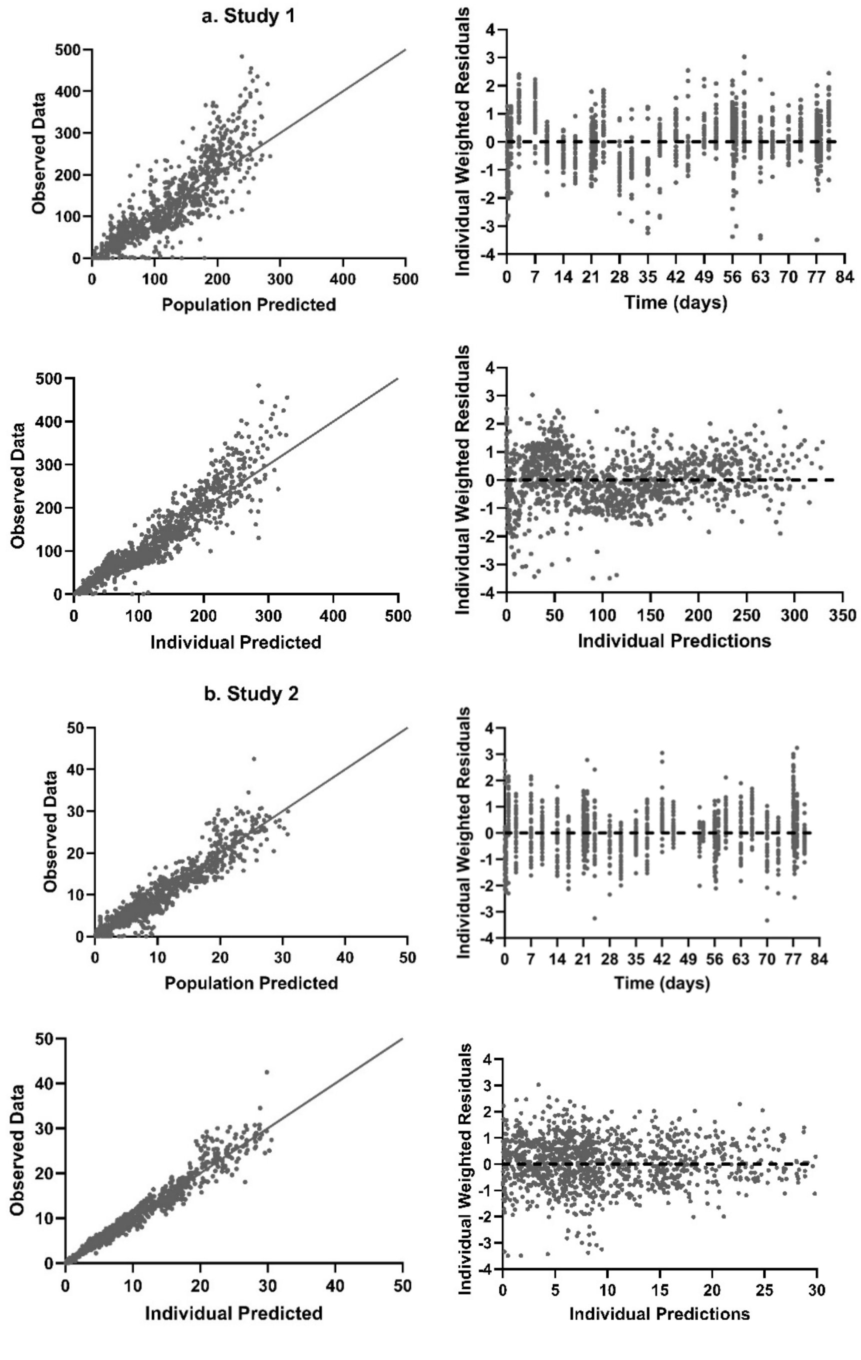

Figure S1. Diagnostic plots for the final population pharmacokinetic model. Observed vs predicted erenumab serum concentrations profiles, Individual weighted residuals vs. time and Individual weighted residuals vs. individual predictions are plotted after subcutaneous administration of erenumab on day 1 of weeks 1, 2, 3, 4, 9, 10, 11 and 12. (**a**) erenumab was administered as monomer at a dose of 10 mg/kg in Study 1 and (**b**) erenumab was administered as aggregates at a dose of 1 mg/kg in Study 2.

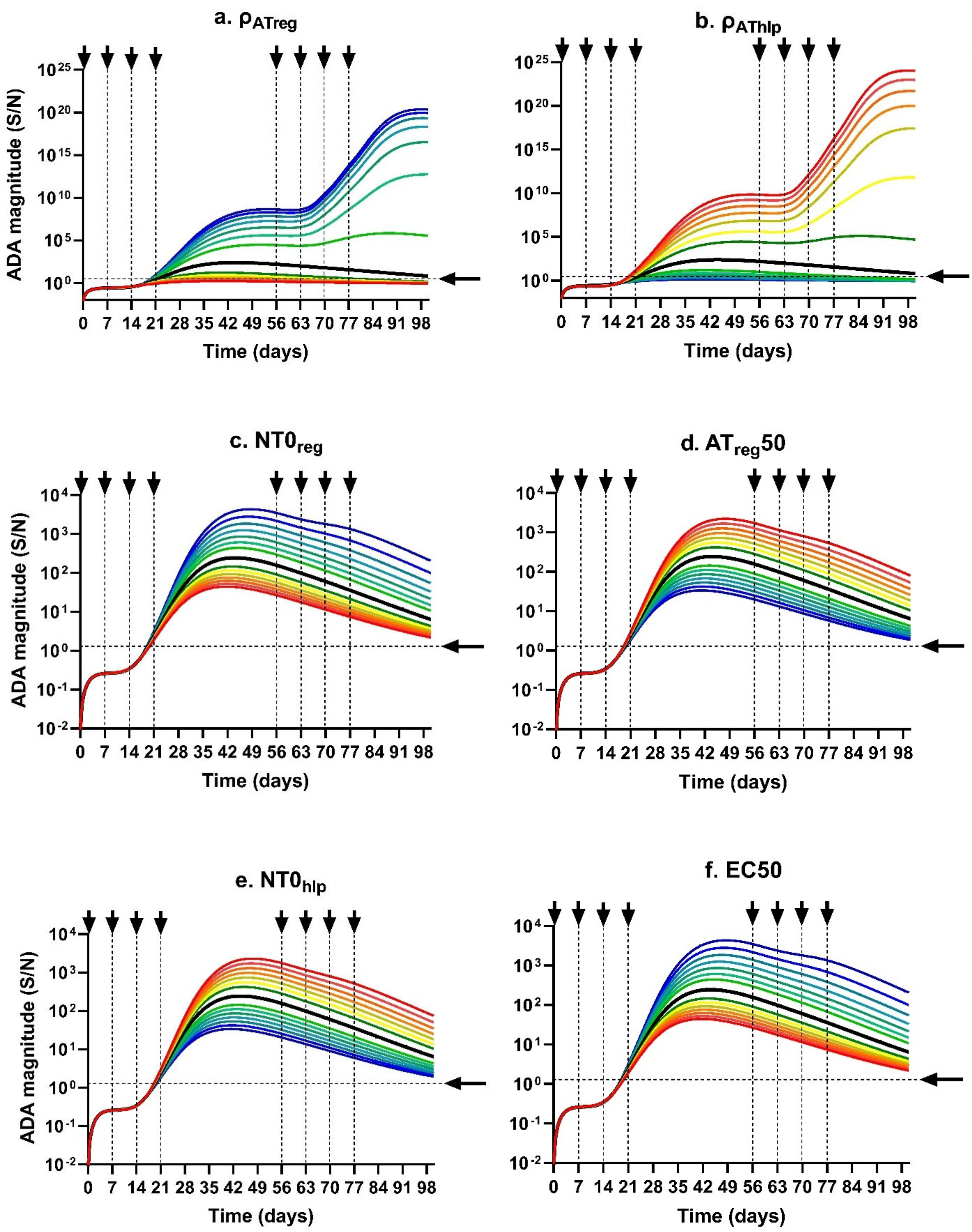
a. ρATreg
b. ρAThlp
c. NT0reg
d. ATreg50
e. NT0hlp
f. EC50
ADA magnitude (S/N)
Time (days)
0 7 14 21 28 35 42 49 56 63 70 77 84 91 98

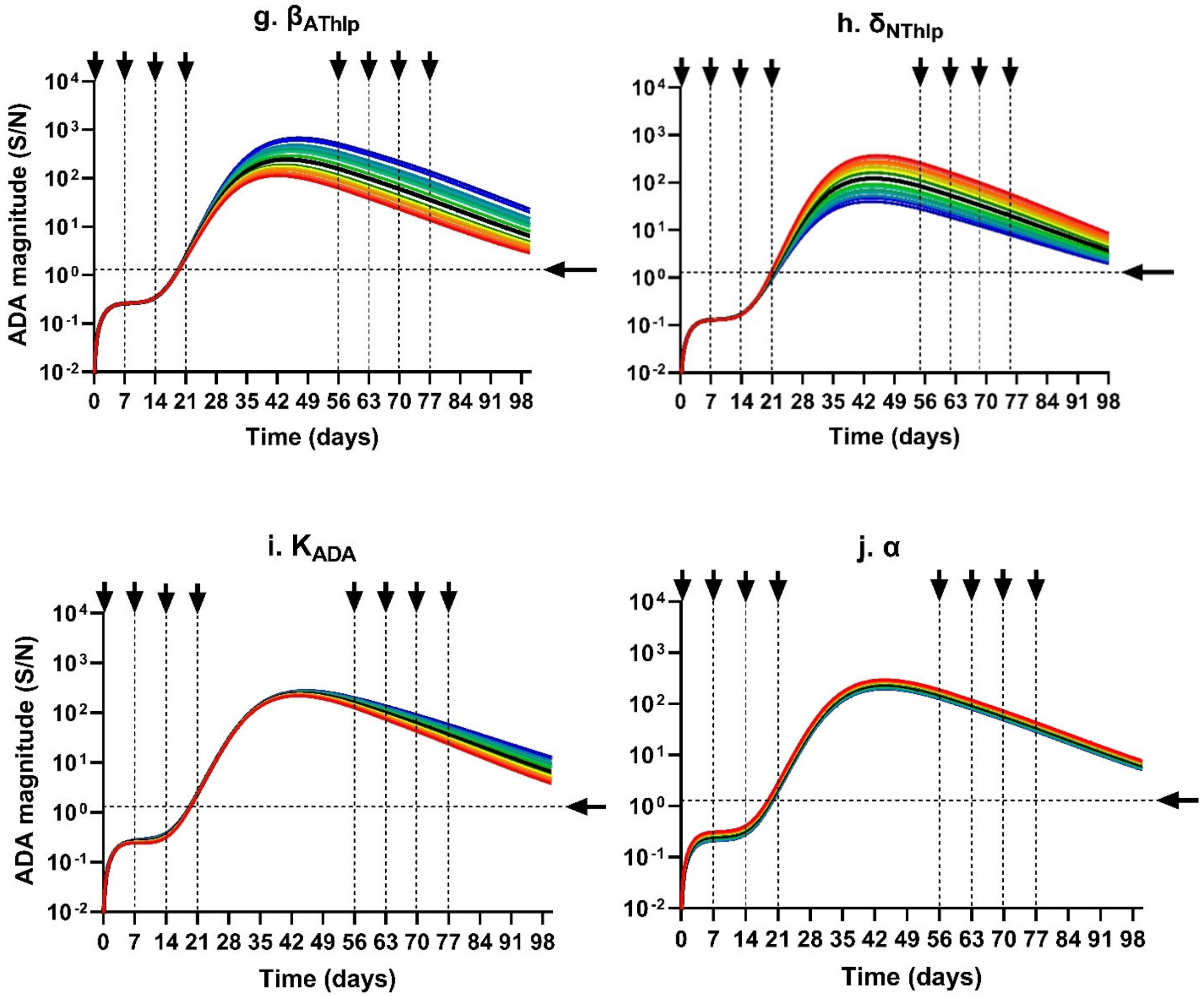

Figure S2. Sensitivity analysis of the effect of select parameters on ADA magnitude time courses. (**a**) Maximum proliferation rate for activated T-regulatory cells (ϱAT_{reg}), (**b**) Maximum proliferation rate for activated CD4$^+$ T-helper cells (ϱAT_{hlp}), (**c**) Initial number of naïve T-regulatory cells ($NT0_{reg}$), (**d**) Number of activated T-regulatory cells required for half-maximal suppression of activated CD4$^+$ T-helper cells ($AT_{reg}50$), (**e**) Initial number of naïve CD4$^+$ T-helper cells ($NT0_{hlp}$), (**f**) Erenumab concentration at which naïve dendritic cell activation rate is 50% maximum (EC50), (**g**) Death rate of activated CD4$^+$ T-helper cells (βAT_{hlp}), (**h**) Maximum activation rate for naïve CD4$^+$ T-helper cells (δNT_{hlp}), (**i**) Elimination rate constant of antibody (K_{ADA}), (**j**) Secretion rate of antibody (α)

Magnitude of ADA with the indicated parameter (ϱAT_{hlp}, $NT0_{hlp}$, $AT_{reg}50$, δNT_{hlp}, α) increased (red) or (ϱAT_{reg}, $NT0_{reg}$, EC50, βAT_{hlp}, K_{ADA}) decreased (blue) 20% from the best-fit value (black).

Horizontal arrows indicate the ADA detection threshold, vertical arrows erenumab dosing times.

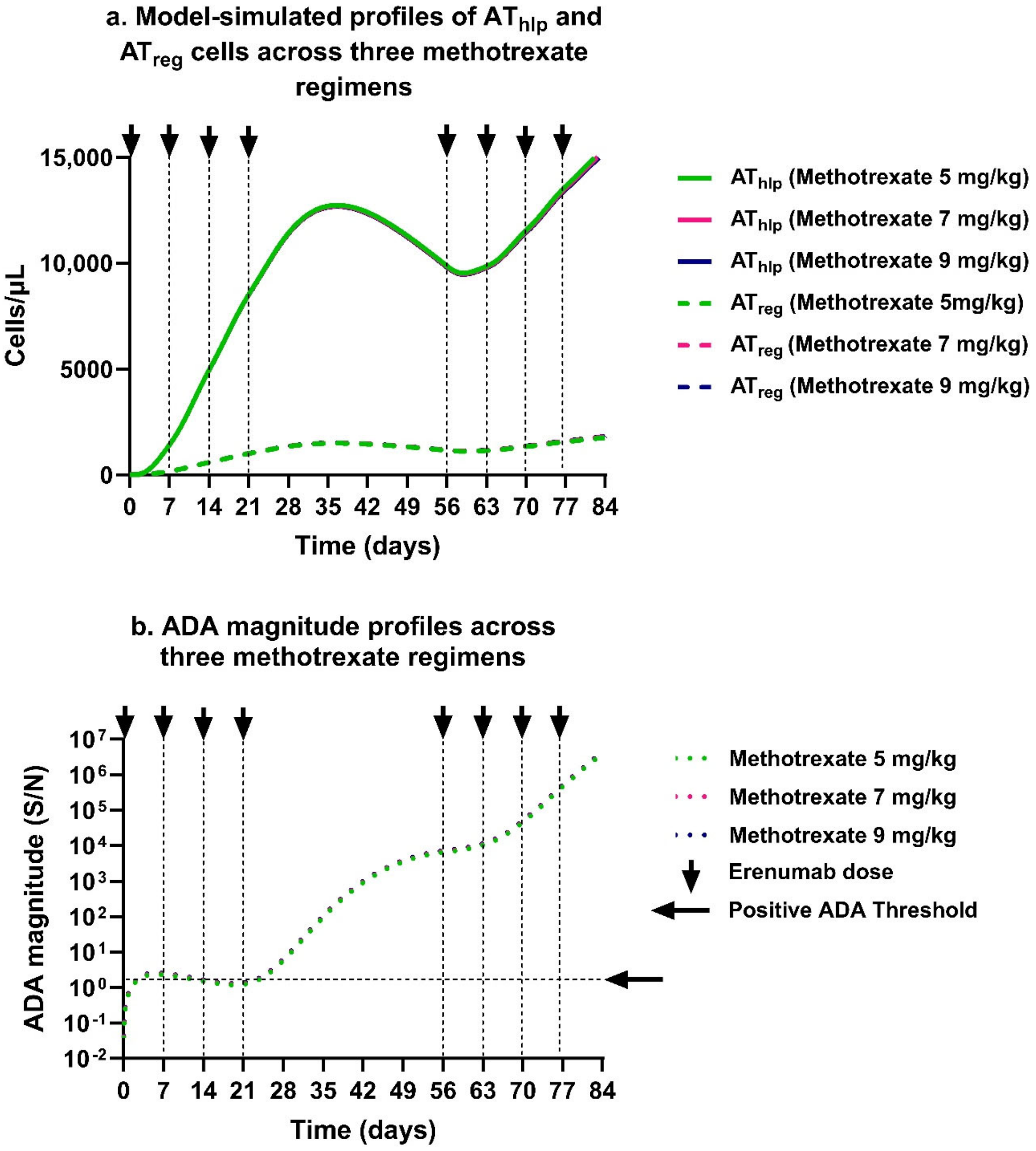

Figure S3. Simulated times courses of activated T cells and ADA magnitude for different methotrexate regimens (I). (**a**) activated CD4+ T-helper cells (AT_{hlp}) and activated T-regulatory cells (AT_{reg}); (**b**) ADA magnitude that received 10 mg/kg erenumab monomer on the first day of weeks 1, 2, 3, 4, 9, 10, 11 and 12 along with methotrexate at 3 dose levels: 5 mg/kg, 7 mg/kg, or 9 mg/kg every week from week 1 to week 7. Profiles in Panel (**a**) are shown on a linear scale and profiles in Panel (**b**) are shown on a logarithmic scale. Positive ADA threshed was set to 2. The arrow represents erenumab dosing time points.

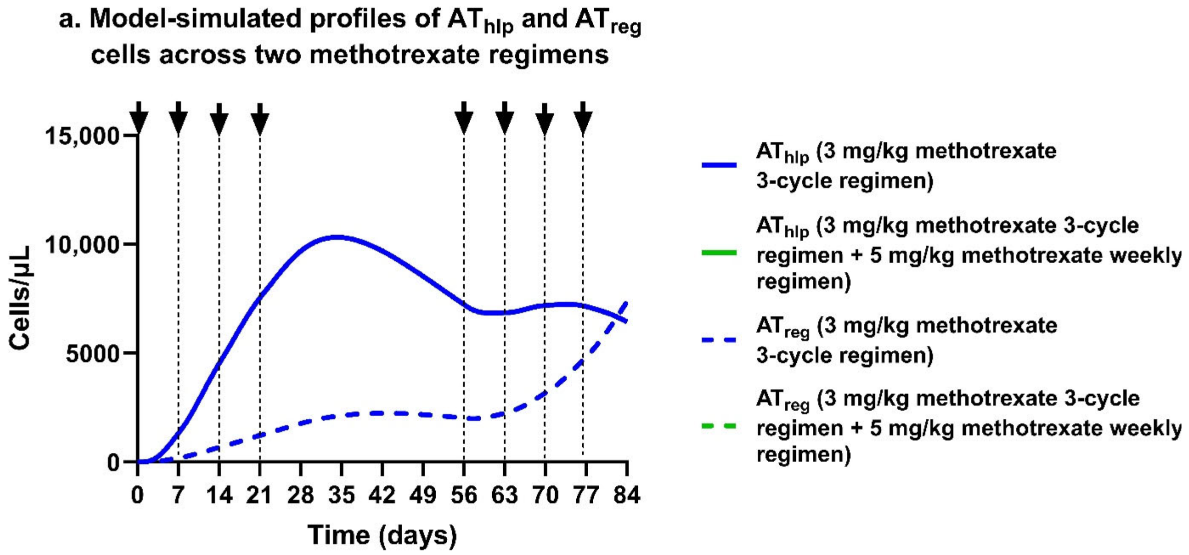

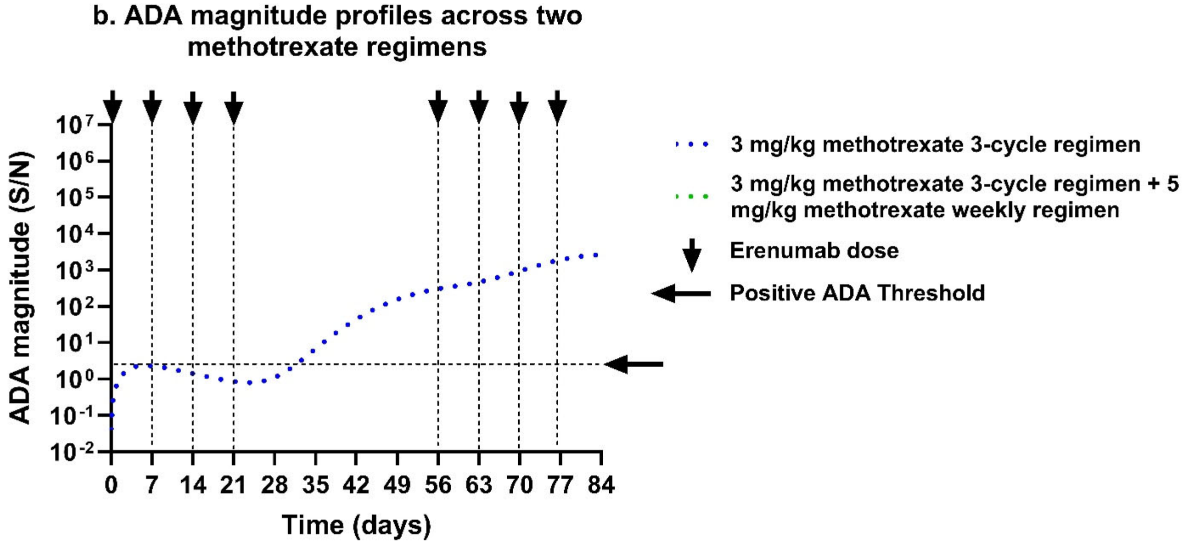

Figure S4. Simulated times courses of activated T cells and ADA magnitude for different methotrexate regimens (II). (**a**) activated CD4$^+$ T-helper cells (AT_{hlp}) and activated T-regulatory cells (AT_{reg}); (**b**) ADA magnitude that received 10 mg/kg erenumab monomer on the first day of weeks 1, 2, 3, 4, 9, 10, 11 and 12 along with 3 mg/kg methotrexate on first, second and third day of the study with and without 5 mg/kg methotrexate every week from week 2 to week 7. Profiles in Panel (**a**) are shown on a linear scale and profiles in Panel (**b**) are shown on a logarithmic scale. Positive ADA threshed was set to 2. The arrow represents erenumab dosing time points.

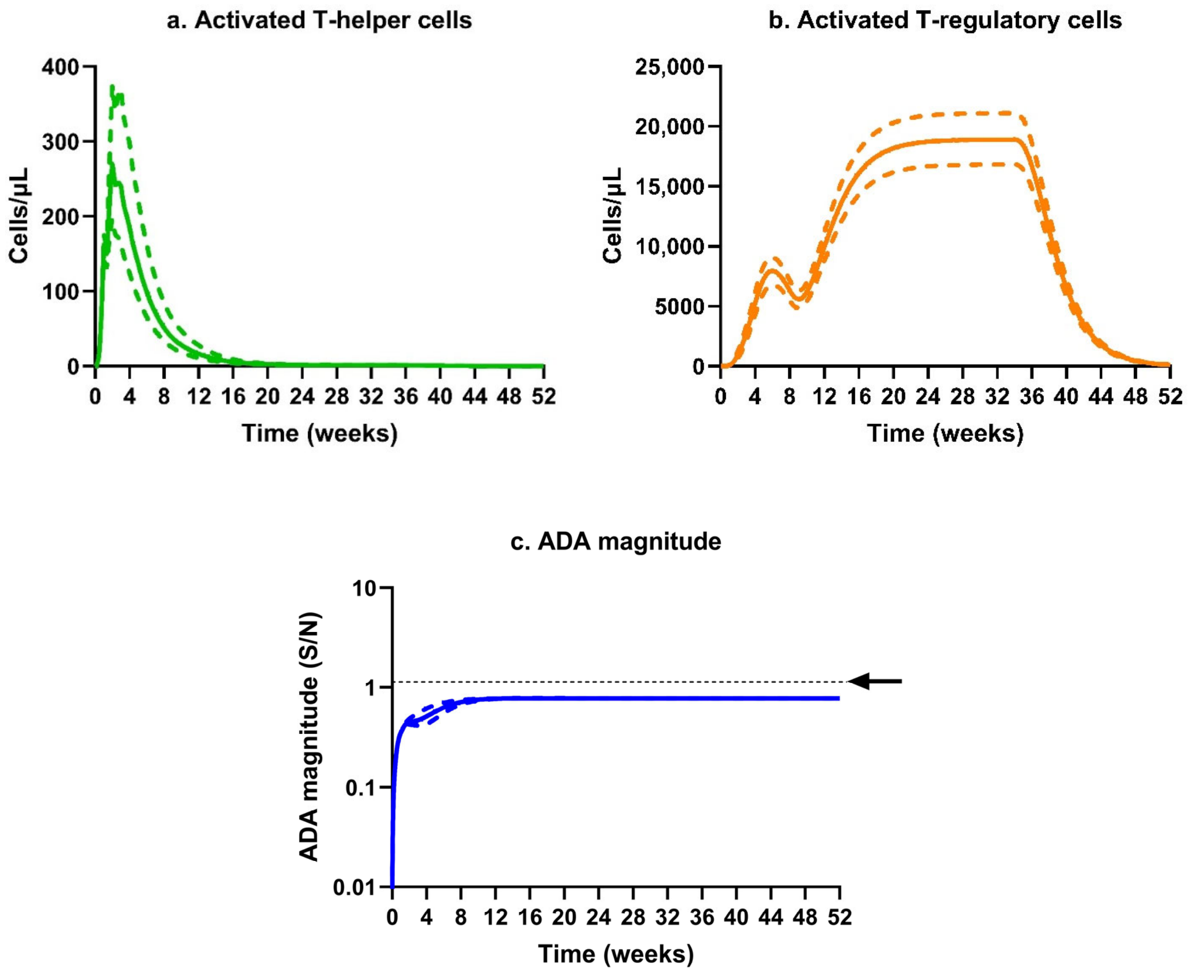

Figure S5. Stochastic simulation of the time courses of T cells, ADA magnitude for a hypothetical chronic toxicology study with erenumab aggregate. (**a**) Activated $CD4^+$ T-helper cells, (**b**) Activated T-regulatory cells and (**c**) ADA-magnitude for a hypothetical 6-month multiple-dose toxicology study in 1000 animals that received 1 mg/kg erenumab aggregate weekly for 6-months in the rechallenge phase. Solid line represents the average values, and the dashed line represents the 95% prediction interval. Cell numbers are shown on a linear scale and ADA magnitude is shown on a logarithmic scale. Positive ADA threshed was set to 1.5 represented by an arrow in Panel (**c**).
